# *In Silico* Identification of Cholesterol Binding Sites in the Transient Potential Receptor Vanilloid 1 (TRPV1) Ion Channel

**DOI:** 10.1021/acs.jpcb.5c08356

**Published:** 2026-04-13

**Authors:** Jacob Morris, Annabel S. J. Eardley-Brunt, Alexander M. Blayney, Carmen Domene

**Affiliations:** Department of Chemistry, 1555 University of Bath, Claverdon Down, Bath BA2 7AY, U.K.

## Abstract

TRPV1 channels are activated by diverse physical and chemical stimuli, including natural products and inflammatory mediators, which often act on their transmembrane regions. Cholesterol, a key lipid in eukaryotic membranes, is known to influence ion channel activity both indirectly, by altering membrane properties, and directly, through specific binding interactions. Using *in silico* molecular docking and site identification by ligand competitive saturation (SILCS), multiple cholesterol binding sites on TRPV1 were systematically identified. These sites were further examined by comparing cholesterol to its stereoisomer epicholesterol, revealing differences in site specificity and interaction patterns. The results highlight structurally persistent cholesterol interaction hotspots that may stabilize channel structure, as well as state-dependent binding motifs potentially involved in functional modulation. This ensemble-based, high-resolution mapping of cholesterol-TRPV1 interactions underscores the need to account for protein conformational flexibility when studying lipid–protein interactions. The findings contribute to a deeper mechanistic understanding of how cholesterol regulates TRPV1 and similar membrane proteins, with broader implications for the modulation of ion channel function by membrane lipids.

## Graphical abstract

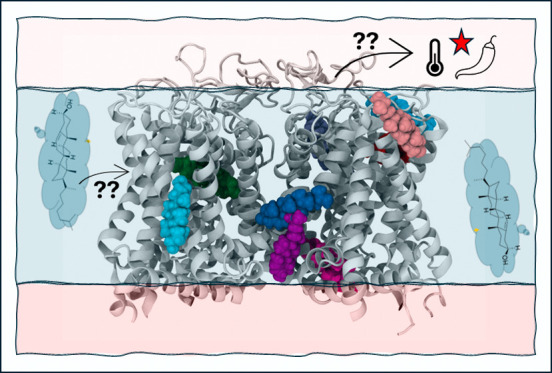

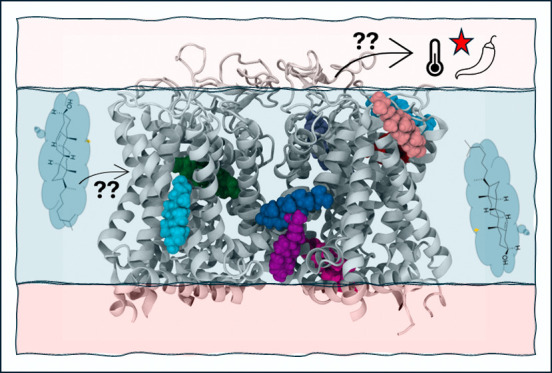

## Introduction

The TRP (Transient Receptor Potential) channels are a superfamily of cation-permeable transmembrane proteins. TRP channels respond to a series of physical and chemical stimuli including temperature, osmolarity, and a variety of natural chemicals allowing the perception of, *e.g.* bitter, sweet and umami tastes.^1^ TRP channels are common in nociceptive (pain sensing) nerve endings, which translate a mechanical, thermal or chemical signal capable of damaging tissue into a feeling of pain via action potentials.^2^ The biological roles of TRPs are broad and varied due to their wide expression and polymodal activity, but are all based on the mediation of the transmembrane flux of cations (most often Ca^2+^ and Na^+^) down electrochemical gradients.^3^ If intracellular Ca^2+^ concentration exceeds basal levels (∼100 nM), many intracellular processes, including metabolism, exocytosis, muscle contraction and RNA transcription, can be initiated.^4^ Through the activity of Na^+^/H^+^ exchangers, Na^+^ entry contributes to intracellular pH regulation, a function essential for many physiological processes such as neuronal growth.^5^

The mammalian TRP channel superfamily consists of 28 channels, split into six subfamilies based on amino acid sequence homology; TRPV (vanilloid), TRPC (canonical), TRPA (ankyrin), TRPM (melastatin), TRPP (polycystin) and TRPML (mucolipin), with one additional family TRPN (no mechanoreceptor potential C), found in invertebrates and fish.^6–8^ The development of cryo-electron microscopy has allowed the determination of TRPV channel structures often in various conformations to atomic resolution.^9–11^ The TRPV (vanilloid) subfamily is named after TRPV1’s most studied ligand, capsaicin, the vanilloid which elicits the burning sensation of chili peppers.^12,13^ TRPV1 is the only TRPV found to be sensitive to vanilloid compounds, and the subfamily is classified only by sequence homology.^13^ Analysis of these structures has revealed that all members of the TRPV subfamily adhere to the same general structure (Figure 1). The tetramers adopt a “domain swapped” structure, meaning that helices S5–S6 of one subunit is packed against S1–S4 of an adjacent subunit. A short extracellular loop joins S1 to S2 in all TRPVs, along with two longer loops connecting S2–S3 and S4–S5, which run parallel to the intracellular membrane interface. N-terminal to S1 is the ankyrin repeat domain (ARD), comprised of six ankyrin repeats and joined to S1 by the membrane-proximal region (ARD-S1). C-terminal to S6 is the TRP domain, a ∼25-amino-acid sequence loosely conserved in the TRP superfamily; its most highly conserved element is the 6-residue TRP box.^9^ It runs parallel to the plasma membrane through a “cradle” formed by ARD-S1, followed by an intracellular C-terminus, which varies in length within the subfamily.^14–19^

**Figure 1:**
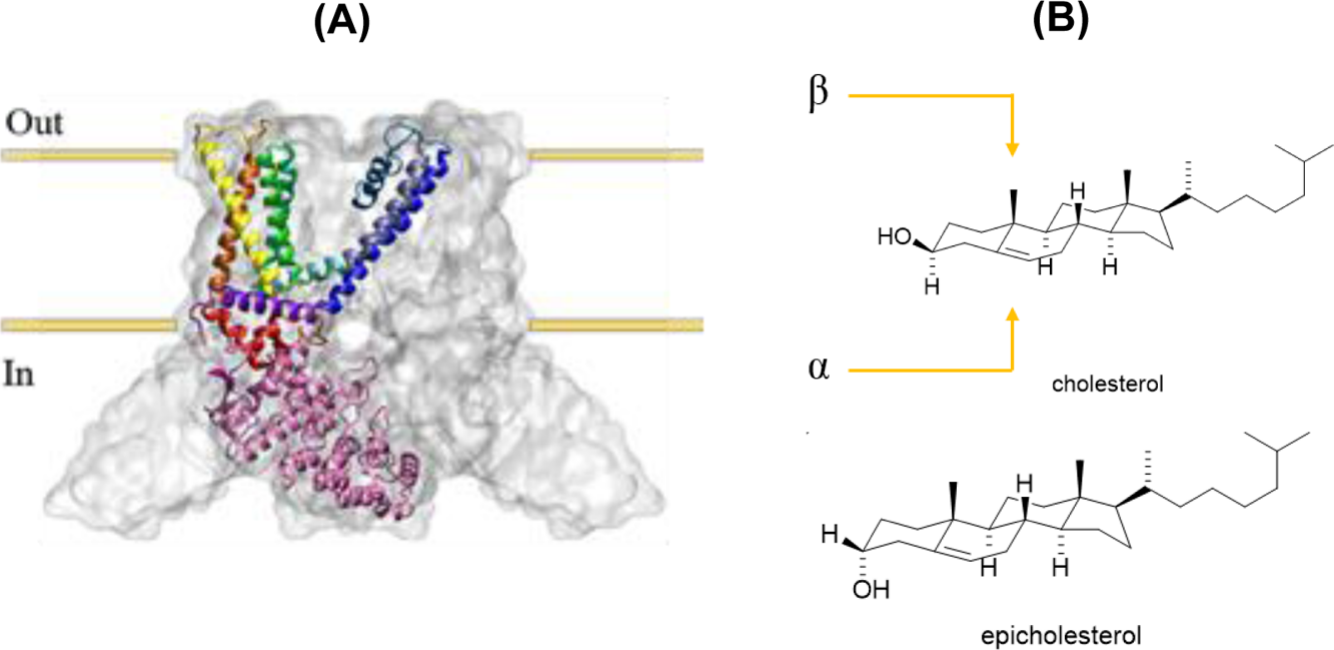
(A) Schematic representation of TRPV1 with each structural element of one monomer colored differently: ARD = pink, ARD-S1 linker = red, S1 = orange, S2 = yellow, S3 = green, S4 = turquoise, S5 = gray, *P* = teal, S6 = blue, and TRP box = purple. The whole channel is displayed as a transparent surface representation, and the plasma membrane location is indicated by the yellow bars, where “Out” is the extracellular matrix and “In” is the intracellular cytosol. (B) Cholesterol and its diastereomer epicholesterol. The ‘rough’ β-face contains two protruding methyl groups, in the case of cholesterol, the α-face is “smooth”. The α-face of epicholesterol contains the axial hydroxyl group.

TRPV1 was the first TRP channel to be cloned, by Julius and colleagues in 1997.^20–22^ TRPV1 is a thermo-TRP, responding to noxious heat (>43 °C), acidic conditions (pH < 6), exogenous ligands and inflammatory mediators, all of which induce a burning sensation upon influx of Ca^2+^.^11,23^ TRPV1 has a high relative Ca^2+^ permeability (P_Ca_/P_Na_ = 9.60) when compared to other nonselective cation channels.^24^ TRPV1 is activated by many natural exogenous compounds including vanilloid food extracts, such as gingerol, eugenol and camphor.^25^ Helices S1–S4 act as a stimulus sensing domain, held rigid by an aromatic core (Y441, Y444, F488, F516, Y554, Y555), while S5–P–S6 is thought to govern channel gating.^26–28^ Annular lipids, which preferentially bind to the surface of membrane proteins, were found to fill gaps between transmembrane helices, and a density representative of phosphatidylcholine was observed to fill a hydrophobic cleft in the S1–S4 region.^28^ It was also determined that depletion in cholesterol concentration substantially reduced TRPV1 potentiation by capsaicin and protons.^29^ However, while cholesterol was essential to TRPV1 expression in cell membranes, it was found to directly exert an inhibitory effect on capsaicin induced TRPV1 activation.^30^ A CRAC (Cholesterol Recognition/interaction Amino acid Consensus) motif is found in the lipid interface of S5, and it was determined that R579, F582 and L585 interact directly with the hydroxyl, aromatic and aliphatic moieties of cholesterol, respectively.^31^

Cholesterol is a major component in eukaryotic cell membranes and plays a central role in regulating bilayer structure and dynamics (Figure 2).^11^ Changes in membrane cholesterol content can alter ion channel function through effects on membrane fluidity, stiffness, and lateral organization, as well as through participation in lipid rafts that influence channel trafficking and localization.^32,33^ Cholesterol and other lipids such as sphingomyelin and gangliosides have also been shown to contribute to ion channel functionality through transport to the plasma membrane.^30,34^ Interactions between cholesterol and transmembrane ion channels have been well studied, and four main mechanisms have been proposed for their regulatory relationship: (i) cholesterol can mediate membrane expression of the channel through trafficking in lipid rafts,^35,36^ (ii) membrane stiffness can be modulated by plasma membrane composition, dictating the energy required for transitions in channel structure,^30^ (iii) cholesterol can interact with other signaling molecules, or receptors, which have a downstream effect on the channel,^37^ and (iv) cholesterol can act directly on the channel to mediate channel activity.^38^

**Figure 2:**
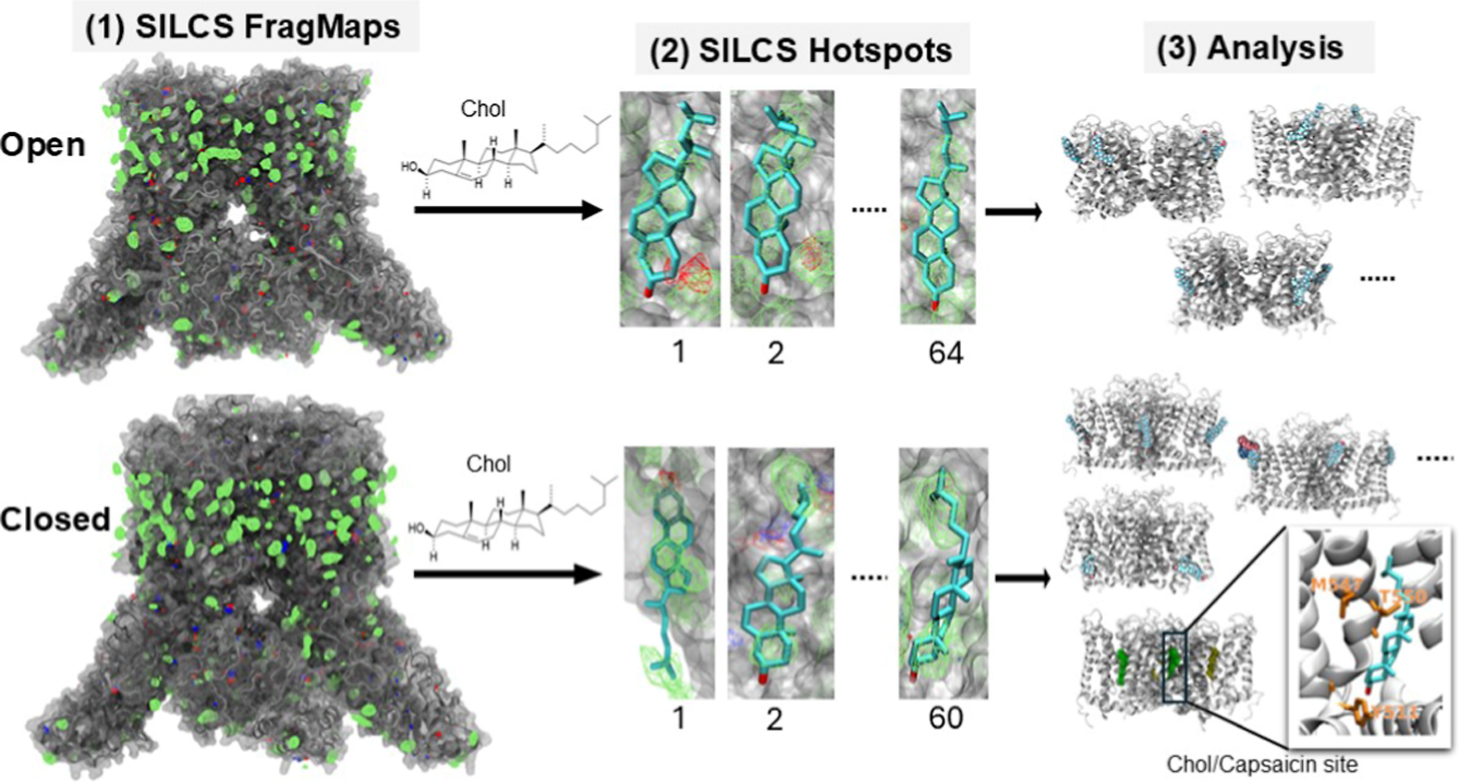
Schematic of the SILCS protocol. SILCS simulations were performed following an established workflow:^1^ FragMaps of TRPV1 in the open and closed conformations are generated.^2^ Hotspot poses are identified in both conformations via SILCS hotspot analysis.^3^ The resulting poses are classified based on their spatial and energetic properties. As an example, cholesterol is shown bound at the capsaicin-binding site in the closed conformation, with relevant protein residues highlighted in orange. Cholesterol adopts a downward-facing orientation, consistent with interactions involving these highlighted residues in the zoomed view.

Early investigations into the interactions between cholesterol and proteins suggested the existence of a simple cholesterol binding motif, the cholesterol recognition amino acid consensus (CRAC) motif.^39,40^ This motif is characterized by the L/V-(*X*)_1–5_-*Y*-(*X*)_1–5_-R/K pattern, where *X* is any linking amino acid residue.^41^ A second cholesterol binding motif which mirrors the CRAC motif, termed CARC motif with pattern R/K-(*X*)_1–5_-*Y*/F-(*X*)_1–5_-L/V, has been also proposed to bind cholesterol with even greater affinity.^40,42,43^ Due to the broad definition of these motifs, they are widespread throughout protein sequences, and therefore are not necessarily indicative of actual cholesterol binding sites.^44,45^ However, the binding of cholesterol to these domains has been characterized for various proteins, including TRPV1.^42,46–50^

One way in which cholesterol has been known to act directly on ion channels is illustrated by the nonidentical effect of epicholesterol (3α-hydroxy-5-cholestene), a diastereomer of cholesterol (3β-hydroxy-5-cholestene), on channel activity, suggesting stereospecific binding of cholesterol.^38,39,51^ Epicholesterol is not naturally abundant in animal tissues but is usually synthesized artificially for research purposes, particularly for studying stereospecific effects of cholesterol on membranes and proteins. Although epicholesterol only differs from cholesterol by the “handedness” of the hydroxyl chiral center (Figure 2), such a structural change appears to have a profound effect on the behavior of the sterol in the membrane. It was suggested that this is due to a difference in hydration of the hydroxyl polar moiety.^52^ This makes epicholesterol extremely useful in determining the specific effects of cholesterol on proteins, as they have very similar effects on membrane properties but their direct effects on proteins differ due to their opposite stereochemistry.^39,53–56^ In the case of inwardly rectifying potassium 2 (Kir2) channels, it was shown that substituting ∼50% of membrane cholesterol for epicholesterol increased current amplitude 2-fold.^39,51^ Evidence suggested that this was due to a direct and specific inhibitory action of cholesterol on the channels, and that cholesterol may even stabilize the closed state of the channels, forming “silent channels” with prolonged states of nonconductance.^51^

In this study, cholesterol binding sites within TRPV1 are investigated *in silico* and compared to epicholesterol binding sites to identify potential sterol binding sites. Cholesterol has been shown to act on ion channels both indirectly and directly.^33,37,39,41^ The wide expression and polymodal activity of the TRPV channels implicate their role in a wide variety of physiological conditions, from nociception and hyperalgesia, to nephrolithiasis, cystic fibrosis and cancer.^38,57–65^ Therefore, understanding the mechanism and effect of direct binding to the TRPV channels is of great pharmaceutical importance. The most significant cholesterol binding sites in TRPV1 in different conformational states are also identified and compared to each other to shed some light into the role of cholesterol in channel function. These results are supported by equivalent data for epicholesterol, used solely as a qualitative control to show that the analysis does not produce sterol-independent binding poses, and to mirror its widespread experimental use as a diagnostic sterol for distinguishing direct, stereospecific cholesterol–protein interactions from indirect membrane-mediated effects, despite its absence from natural membranes, and are contextualized within the broader picture of TRPV1 channel modulation by lipids and other small-molecule ligands.

## Computational Methods

### Classical Docking

#### System Preparation

PDB files for TRPV1 (3J5P, 3J5Q, 3J5R, 5IRX, 5IRZ, 5IS0) were obtained from the RCSB Protein Data Bank.^21,22,66^ Missing loops 503–507 [3J5P/Q/R] were modeled with the protein builder feature of the Molefacture modeling extension in VMD, before being refined using ModLoop, an online extension of the MODELLER software.^19,67^ The PDB file for cholesterol was obtained from a β-cryptogein-cholesterol complex in the RCSB PDB (1LRI), and the epicholesterol PDB was created in VMD using Molefacture.^68,69^ A summary of the systems used in this study is presented in Table 1.

**Table 1:** Systems Considered in this Study

System	PDB ID	Resolution (Å)	Species	Conformation
TRPV1	3J5P	3.275	Rattus norvegicus	closed
	3J5Q	3.80		open
	3J5R	4.20		open
	5IRX	2.95		open
	5IRZ	3.28		closed
	5ISO	3.43		closed

### Docking Protocol

Hydrogens were added to both ligand and protein, and Gasteiger charges calculated in AutoDockTools before nonpolar hydrogens were merged. The hydroxyl hydrogen was added to cholesterol/epicholesterol in AutoDock4; all nonpolar ligand hydrogens were considered merged.^69^ The grid box was calculated using the AutoGrid software in AutoDockTools, with dimensions of 114 × 64 × 90 points and a grid spacing of 0.725 Å. This grid box was centered on the protein encompassing two out of the four monomers, the minimum unit that contains all possible binding sites.

The docking was performed with AutoDock4, using a Lamarckian Genetic Algorithm with an initial population of 150 randomly placed individuals, a maximum number of 2.5 million energy evaluations, a maximum of 27,000 generations, mutation and crossover rates of 0.02 and 0.80, respectively, and an elitism value of 1. AutoTors (as implemented in AutoDock Tool Kit) was used to define the ligand torsional degrees of freedom. Clusters were calculated by AutoDock4 using an RMSD tolerance of 2 Å.^70,71^ This was repeated 300 times for each docking problem.

Clusters that consisted of at least 2% of the total docked conformations were selected as significant for analysis and are considered as representative of the whole docking procedure; the rest of the solutions were discarded. The docked sterol clusters in the same binding pocket were classified again into groups, sharing most of their interacting residues. We consider a protein residue to interact with a sterol pose if any heavy atom of the residue is within a distance corresponding to the sum of the van der Waals radii of the two atoms. Using commonly accepted van der Waals radii (*e.g.*, ∼1.7 Å for carbon, ∼1.55 Å for nitrogen, ∼1.52 Å for oxygen), this corresponds to approximate heavy-atom contact distances of ∼3.4–4.0 Å for typical carbon–carbon and carbon–heteroatom pairs.

### Site Identification by Ligand Competitive Saturation Approach

The SILCS methodology^72^ was employed as an alternative to classical docking approaches which typically treat the receptor as rigid. In this study, SILCS was applied to the TRPV1 channel to identify cholesterol-binding hotspots which are common in membrane proteins such as TRP channels. The SILCS workflow was applied to the equilibrated bilayer and comprises three principal stages:^1^ GCMC-MD (Grand Canonical Monte Carlo–Molecular Dynamics) simulations for solute and water sampling,^2^ computation of free energy FragMaps and overlap coefficients (OCs), and^3^ Monte Carlo (MC) ligand sampling using FragMaps to derive ligand grid free energies (LGFEs). A schematic of the SILCS workflow is shown in Figure 2.

SILCS generates functional group free energy maps (“FragMaps”) through molecular dynamics-Monte Carlo (MD/MC) simulations that account for protein flexibility and solvent effects, thereby providing detailed information for subsequent ligand docking. The method can accommodate multiple ligand rotamer and protonation states, as well as chiral configurations, helping to address the sampling challenges often encountered in modeling ligand–protein interactions.

Using the oscillating chemical potential grand canonical Monte Carlo/molecular dynamics (GCMC/MD) protocol, receptor FragMaps were generated to assess fragment affinities across the channel, including regions at the protein–membrane interface. These FragMaps were subsequently used to model cholesterol binding affinities, providing a structure-based framework to investigate cholesterol-TRPV1 interactions.

SILCS is certainly more computationally intensive than traditional docking because it requires extensive molecular dynamics or Monte Carlo simulations of the target protein in mixed solvent to generate FragMaps. This is the reason only two PDBs, 3J5P and 3J5Q, were selected for these calculations, where the protein is in the closed and open conformations respectively. Despite this increased cost, SILCS offers improved accuracy relative to traditional docking as it accounts for protein flexibility and solvent effects through precomputed FragMaps. By using these two structures, we aim to capture the conformational extremes relevant to ligand binding, while keeping the computational cost of FragMap generation manageable.

The simulation box dimensions were set to 159 Å in the *X* direction, 170 Å in the *Y* direction, and 170 and 161 Å along the *Z* axis for the closed and open systems respectively. For minimization and equilibration, the standard CHARMM-GUI membrane builder GROMACS protocol was used which includes 5000 steps of energy minimization using the descent method with constraints applied on all non-hydrogen atoms. This is followed by a short MD relaxation phase of 50 ps initialized with a 1-fs time step. Temperature was maintained at 303.15 K using the Berendsen thermostat. A subsequent 325 ps MD simulation had the pressure maintained at 1 bar using the Berendsen pressure coupling and the time step was increased to 2 fs. All position restraints were gradually turned off during the relaxation phase.

Solute mapping was performed using a hybrid GCMC-MD framework that modulates chemical potential to enhance sampling of water and probe solutes within the lipid environment.^73^ The probe set consisted of eight small solutes (benzene, propane, methanol, formamide, dimethyl ether, methylammonium, imidazole and acetate), each representing distinct chemical functionalities (*e.g.*, hydrophobic, hydrogen-bond donors/acceptors, aromatic groups etc.), introduced at 0.25 M concentration in a 55 M aqueous background. These probes and water were used to generate 14 fragmaps (Table S1). An example of these fragmaps is shown in Figure 2.

All-atom MD simulations were performed under NPT conditions at 303 K and 1 atm. Simulations employed a 2 fs time step with periodic boundary conditions. Electrostatics were treated with the particle mesh Ewald method, using a 12 Å real-space cutoff and a PME grid with a 10^–4^ tolerance.^74^ Temperature control was maintained using the Nose–Hoover thermostat^75,76^ at 300 K with a collision frequency of 1 ps^–1^, and pressure was regulated at 1 atm using the Parrinello–Rahman barostat,^77^ while the time constant for pressure and temperature coupling was 1 ps. The LINCS algorithm^78^ was used to maintain bond geometries with a coupling interval of 100 steps. An additional 200 ns of unrestrained NPT equilibration was carried out to ensure full bilayer relaxation prior to SILCS sampling. Following this, ten independent GCMC-MD simulations were performed for each target, initiated from distinct solute and water distributions, yielding an aggregate of 1 μs of production MD per system. GCMC-MD simulations were conducted sequentially for a total of 126 cycles per replica, comprising 26 equilibration cycles followed by 100 production cycles. Each cycle included 200,000 GCMC steps, 5000 steps of steepest-descent minimization, 100 ps of equilibration MD, and 1 ns of production MD. Simulation snapshots were saved every 10 ps. Protein backbone Cα atoms were restrained using a force constant of 0.12 kcal mol^–1^ Å^–2^.

All simulations were performed using GROMACS 2018.3 and SILCS software suite, Version 2021 (SilcsBio, LLC). Protein and lipid parameters were taken from the additive CHARMM36m^79^ and CHARMM36 force fields,^80^ respectively, while probe solutes and ligands were described using the CHARMM General Force Field (CGenFF).^81^

### FragMap, GFE (Grid Free Energy), and Overlap Coefficient Computations

Trajectories from the 10 GCMC-MD replicas were used to compute voxel-based occupancy maps for each solute atom type, binned in 1 Å slabs along the bilayer normal (*Z*-axis) and covering the entire in-plane surface. Occupancies were normalized by solute concentration and converted into free energy maps using Boltzmann transformation, yielding grid-based free energies (GFE FragMaps).^82^ The voxel average across the system was zeroed, providing absolute GFE values. To evaluate FragMap convergence, overlap coefficients (OCs) ranging from 0 to 1, were computed between the first and last five trajectories for each solute type. High OC values (>0.6) support consistent and converged sampling.^72^ Table 1S in the Supporting Information records OC values for the simulations of the closed and open conformation systems.

#### SILCS-Hotspots^83^ Docking

Cholesterol and epicholesterol binding pockets were identified by dividing the system into 14.14 × 14.14 × 14.14 Å^3^ boxes and performing ligand Monte Carlo sampling within each box. Each sampling run consisted of 50,000 MC steps at 300 K, followed by 40,000 additional steps in which the temperature was ramped down from 300 to 0 K. This procedure was repeated 1000 times per box. The resulting configurations were then clustered using a 3 Å center-of-mass radius to obtain a reduced data set, with only ligands exhibiting a ligand GFE below −3.5 kcal/mol included in the clustering. A final clustering step with a 4 Å radius was applied, yielding a set of hotspots around TRPV1 where cholesterol is expected to bind. Examples of the final docking poses can be found in Figure 2.

## Results & Discussion

### Classical Docking: TRPV1 Closed Conformations (PDB 3J5P, 5IRZ, and 5IS0)

Analysis of three closed-state structures, 3J5P, 5IRZ, and 5IS0, revealed shared cholesterol and epicholesterol binding patterns, consistent with structural heterogeneity across these conformational snapshots.

The closed-state structure (PDB 3J5P, 3.4 Å resolution^22^) revealed three major cholesterol binding sites. The primary site, Site 1a, is located between helices S1 and S2 at the extracellular membrane interface and accounts for 38% of docked conformations (Table S2). A second prominent cluster (Site 1b) is found in a hydrophobic cleft at the cytosolic boundary between two adjacent subunits, corresponding to the known vanilloid binding site reported in previous studies.^84^ The third significant cholesterol binding site (Site 1c) resides in the transmembrane pocket formed by S3, the S4–5 linker, and the TRP domain. A minor site (Site 1d) was also identified at the cytosolic interface between two subunits.

Epicholesterol showed three analogous clusters in the 3J5P structure. One cluster (Site 2a) binds S1 and S2 similarly to Site 1a (Table S3) reflecting minimal influence of hydroxyl group chirality on binding. Another cluster (Site 2b) corresponds to the vanilloid binding site but with epicholesterol oriented opposite to cholesterol, accounting for 31% of docking predictions. The third binding site (Site 2c) lies horizontally between the S2–3 and S4–5 linkers, stabilized by a hydrogen bond between the epicholesterol hydroxyl and Ser512 side chain oxygen.

In the closed TRPV1 structure at 3.28 Å resolution (PDB 5IRZ),^29^ the vanilloid binding site (Site 3a) dominates cholesterol binding with 88% occupancy. This site involves interactions with S3, S4, the S4–5 linker, the TRP domain of one subunit, and S5 and S6 of the adjacent subunit. The conformation analogous to Site 1a, Site 3b, accounts for only 10% of docked cholesterols, indicating conformational differences between these two closed structures. Site 3b is closer to the cytosolic interface.

Epicholesterol binding in 5IRZ is even more concentrated at the vanilloid site (Site 4a) with 97% occupancy. This shift likely reflects the loss of a stabilizing hydrogen bond present with cholesterol in Site 3b.

For the antagonistically closed TRPV1 structure crystallized with capsazepine (PDB 5IS0), 56% of docked cholesterols occupy Site 5a between S1 and S2,^29^ nearly identical to Site 1a. The next largest cluster (Site 5b, 17%) is found in the pocket formed by S2, S3, and the linker. A minor cluster at the vanilloid site (Site 5c) holds 12%. A further minor site (Site 5d) overlaps with Site 3b.

Epicholesterol preferentially binds between S1 and S2 (Site 6a) in 5IS0, accounting for 66% of clusters, closely mirroring Site 5a. A secondary epicholesterol site (Site 6b) near the cytosolic interface is stabilized by hydrogen bonding with Asn437. Three additional minor epicholesterol sites were observed: Site 6c, Site 6d in the vanilloid site, and Site 6e.

Overall, the vanilloid pocket emerges as a recurrent high-affinity binding site for cholesterol and epicholesterol, particularly in 5IRZ. Meanwhile, the S1–S2 interface is frequently occupied in both 3J5P and 5IS0. Binding patterns are modulated by differences in conformation, hydroxyl orientation, and hydrogen bonding capacity, underlining the dynamic and context-dependent nature of sterol interactions with TRPV1.^23^

### Classical Docking: TRPV1 Open Conformations (PDB 3J5Q, 3J5R, 5IRX)

Three cryo-EM structures of TRPV1 in open conformations reveal distinct but partially overlapping cholesterol and epicholesterol binding profiles. In the fully open, toxin-bound TRPV1 structure (PDB 3J5Q, 3.8 Å resolution), cholesterol predominantly docks at a site designated Site 7a, located on the opposite face of S4 from the vanilloid binding pocket (Table S2).^30^ In contrast, the dominant epicholesterol binding site in this structure, Site 8, lies at the interface of S1 and S4 from one subunit and S5 of an adjacent subunit (Table S3). The orientation of epicholesterol is stabilized by a hydrogen bond between its hydroxyl group and the backbone carbonyl of Phe439.

A second open conformation, obtained in the presence of capsaicin (PDB 3J5R, 4.2 Å resolution), displays a different cholesterol binding profile. Here, cholesterol preferentially occupies Site 9a, located parallel to the membrane along S2 and the S2–S3 linker.^23^ Two additional, minor cholesterol binding modes were identified: Site 9b, at the interface of S2–S4 and the TRP domain, and Site 9c, at the intracellular interface of adjacent subunits, between S4–S5 and S6. Epicholesterol also binds to this conformation, with the most prominent site, Site 10a, residing in the S2–S3 region. This site differs from the corresponding cholesterol site (Site 9a) by a hydrogen bond formed between the hydroxyl group of epicholesterol and the backbone carbonyl of Phe507. Two minor epicholesterol binding sites were observed: Site 10b at the intracellular pore interface, involving residues from the S4–S5 linker and S6, and Site 10c in the hydrophobic cleft between S5 and the pore helix of one subunit and S6 of a adjacent subunit.

A third open-state structure, also bound to RTX/DkT*x* (PDB 5IRX), further supports the preference of cholesterol and epicholesterol for sites near S4 and S5. In this structure, the main cholesterol binding site, Site 11a, closely resembles Site 4a in the closed state but is shifted closer to the extracellular leaflet.^30^ A second cholesterol site (Site 11b) is found between extended S5–S6 regions of adjacent subunits, while a small proportion of docked cholesterol molecules occupy the vanilloid binding pocket (Site 11c). Epicholesterol docking in 5IRX closely mirrors the pattern seen in 3J5Q, with 82% of the docked conformations localized to Site 12a, an intersubunit site involving S1 and S4 from one subunit and S5 from the adjacent one. This site differs from the cholesterol-bound Site 11a mainly in the orientation of the hydroxyl group. In epicholesterol, the hydroxyl group points toward S1, forming a stabilizing interaction with Phe438, while in cholesterol it points toward S4, engaging Tyr555. A second major epicholesterol binding site, Site 12b, is located at the S5–S6 interface between adjacent subunits and corresponds closely to the cholesterol site 11b.

Therefore, across the three open conformations of TRPV1 (PDBs 3J5Q, 3J5R, and 5IRX), several recurring binding sites for cholesterol and epicholesterol emerge, particularly in regions involving the S4–S5 interface and intersubunit contacts: the S4–S5 interface on the *cis*-subunit involves residues on the intracellular side of S4 and S5. This site consistently hosts cholesterol (Sites 7a and 11a) and epicholesterol (part of Site 12a). Located opposite the vanilloid binding pocket, it appears favored in fully open toxin-bound conformations (3J5Q and 5IRX).

An intersubunit site bridging S1, S4 (cis), and S5 (trans) is a preferred binding region for epicholesterol in both 3J5Q and 5IRX (Sites 8 and 12a). Key interacting residues include Phe438 on S1, Met552 and Thr556 on S4 from one subunit, and Phe582, Leu585, Val586, and Phe589 on S5 from the adjacent subunit. The orientation of the epicholesterol hydroxyl group and its hydrogen bonding partner (Phe439 vs Phe438) varies slightly between these structures, but the site remains topologically consistent.

At the extracellular S5–S6 and pore helix interface, on the *trans*-subunit, both cholesterol and epicholesterol bind an intersubunit site involving residues on S5, S6 and the pore helix (Sites 11b and 12b). This binding site is observed in 5IRX for both sterols and likely reflects a structurally conserved feature of the open channel.

Finally, the S2–S3 region facing the intracellular membrane, observed mainly in the capsaicin-bound 3J5R structure, hosts cholesterol and epicholesterol near residues Tyr495, Phe496, Arg500, and the segment Ser502–Ser510 (Sites 9a and 10a). While these sites are less prominent in other structures, this region appears permissive for both sterols, possibly due to increased exposure in this conformation.

These shared binding patterns suggest that S4–S5 and intersubunit regions function as privileged cholesterol-sensing elements in the TRPV1 open state, likely contributing to the sterol-dependent modulation of channel function.

Comparing the closed and open TRPV1 structures, cholesterol preferentially binds at the vanilloid binding site and the S1–S2 extracellular interface, with epicholesterol showing similar site preferences but with altered orientations and hydrogen bonding patterns due to its hydroxyl stereochemistry. The vanilloid site’s high occupancy supports cholesterol’s potential role in modulating TRPV1 activation and ligand binding. Minor sites, including the S2–S3 linker and interfaces between subunits, may contribute to allosteric regulation or transient interactions.

The differences in cholesterol and epicholesterol binding orientations and site occupancy suggest the importance of stereochemistry in cholesterol’s modulatory effects on TRPV1. These findings can inform future experimental studies on lipid modulation of TRP channels and aid in the design of cholesterol-mimicking modulators for pain management.

### Competitive vs Allosteric Inhibition of TRPV1

Previous studies have shown that membrane cholesterol is required for maintaining TRPV1 at the plasma membrane, as cholesterol depletion reduces TRPV1 levels in membrane fractions.^30^ Direct binding of cholesterol to TRPV1 has been shown to inhibit capsaicin-induced activation.^29,30,35,38^ Direct binding of cholesterol to TRPV1 was demonstrated by showing that epicholesterol did not elicit the same inhibitory action, suggesting a stereospecific cholesterol binding mode.^31^ Direct binding of cholesterol could inhibit capsaicin activation of TRPV1 either competitively, by binding to the same active site, or allosterically, by binding to another site, reducing the open probability of the channel. Consistent with this, a cryo-EM study revealed a putative cholesterol molecule stabilizing an antagonist-bound conformation of human TRPV1, supporting a direct structural role for cholesterol in modulating channel inhibition.^85^

Capsaicin binds to TRPV1 in what is now known as the vanilloid binding site. This site is made up of multiple residues pointing into the hydrophobic cavity formed by S3, S4, the S4–5 linker and the TRP domain of one subunit and the pore domain (S5 and S6) of an adjacent subunit.^31,84,86^ This binding site has been shown to be occupied by annular lipids in the absence of vanilloids in the cryo-EM structures of TRPV1.^28,29^ The presence of cholesterol in the vanilloid binding site was abundant in the docking results for the TRPV1 structures in closed conformations (sites: 1b, 2b, 3a, 4a, 5c, 6d), accounting for 44% of dockings in these structures. While this may be consistent with competitive cholesterol binding in the vanilloid binding site, there was found to be no significant difference between the binding of cholesterol and epicholesterol to this site. Unfortunately, due to the large standard error (∼2.6 kcal mol^–1^) associated with the semiempirical scoring algorithm implemented in AutoDock4, comparisons of the mean binding energy of cholesterol and epicholesterol in the vanilloid binding site showed no significant difference.^29^ The vanilloid binding site was much less abundant in our data when docked with TRPV1 in open conformations, suggesting that the narrower pocket geometry characteristic of the open state has reduced compatibility with cholesterol.^71^

This study highlights several potential allosteric binding sites for cholesterol, some of which have been previously identified as TRPV1 annular lipid binding sites.^87^ This includes:^1^ a binding ridge at the extracellular termini of S1 and S2, representative of sites 1a, 2a, 11a and 12a,^2^ a binding pocket at the interface of the pore forming domains of adjacent subunits, representative of sites 6c, 10c, 11b and 12b, and^3^ another site bound to S1 and S2, closer to their cytosolic termini, representing sites 3b, 4b, 5d, and 6b. However, once again, none of these sites display a significant preference for cholesterol binding over epicholesterol. Only subtle differences can be noted between the docking of cholesterol and epicholesterol to TRPV1. For example, the different hydrogen bonding interactions evident for 5IRZ (Site 3b), 3J5Q (7a vs 8), 3J5R (9a vs 10a) and 5IRX (11a vs 12a), may play an important role in TRPV1 channel gating, however, these docking data are insufficient to establish a mechanism of the action of bound cholesterol on TRPV1.

While classical docking provides an initial overview of potential cholesterol binding sites, its treatment of the protein as largely rigid and the limited ligand flexibility can reduce predictive accuracy; to address these limitations, we employed SILCS, which more fully accounts for protein conformational variability and energetically favorable sterol–protein interactions.

### The Site Identification by Ligand Competitive Saturation Approach

Using cholesterol as the ligand, SILCS-hotspots docking was performed on fragment-based maps (FragMaps) generated for TRPV1 in both open and closed conformations. TRPV1 functions as a tetramer, explaining the repeated observation of identical binding solutions across three to four subunits (Table 2). The results revealed several residue clusters coinciding with previously identified cholesterol-recognition motifs, including CRAC, CARC, and CRAC-like sequences within the transmembrane domain (Figures 4 and 6). Notably, residues such as Val440, Ser483, Tyr444, Tyr487, and Ile573 appeared repeatedly across these clusters, aligning with key positions in known cholesterol-binding sequences.

**Table 2:** Clusters of Amino Acid Residues Mediating Cholesterol Interactions in the Open and Closed Conformations of the TRPV1 Ion Channel^a^

Group	# Protein Subunits Occupied	Residues	Stereospecificity
OPEN TRPV1			
1	4	Ile573, Leu577, Met581, Cys578 (S5), Leu669 (S6)	false
2	4	Asn437, Val440, Tyr444 (S1), Ile479, Ser483, Tyr487 (S2)	false
3	4	Val482 (S2), Leu524, Val527, Val528, Phe531	false
4	2	Ala450, Tyr454 (S1), Phe473, Gly477 (S2)	true
5	2	Arg409, Hse410 (LD), Lys571, Arg575 (S5), Lys688, Ile689 (S6), Glu692, Asn695, Ile696, Leu699 (TRP)	true
6	3	Tyr631, Ser632, Leu635 (PH), Phe649, Thr650, Ala657, Ile660 (S6)	false
CLOSED TRPV1			
1	4	Val440, Tyr441, Tyr444 (S1), Thr476, Ile479, Leu480, Ser483, Gly484, Tyr487, Phe488 (S2)	false
2	2	Tyr511, Ser512, Leu515 (S3), Phe543, Ala546, Met547, Thr550 (S4), Ile573, Phe591 (S5), Leu662, Ala665(S5), Leu699 (TRP)	true
3	3	Val482, Val486 (S2), Leu524, Val528 (S3)	false
4	2	Phe429, Arg432, Ile433, Tyr435, Phe436, Phe439 (S1), Phe712, Cys715 (TRP)	false
5	2	Gln560, Gln561, Ile564, Tyr565, Met568, Arg579 (S5), Met677, Leu681, Glu684 (S6), Lys694, Lys698 (TRP)	true

a Clusters of overlapping residues were identified by analyzing 64 and 60 cholesterol-protein clusters of interactions from the open and closed conformations respectively derived from SILCS FragMaps and cholesterol docking. Each cluster corresponds to a group of residues of the protein where one or more atoms of an amino acid is at 3 Å of a cholesterol molecule. Only residues shared across all entries within each cluster are reported, representing regions potentially mediating cholesterol binding. The Occurrence column indicates the number of protein subunits in which independent cholesterol-binding events were observed. Residues listed before a segment annotation (seg) belong to that transmembrane segment. Segment labels (S1–S6) denote the six transmembrane helices of TRPV1, where S1–S4 form the voltage-sensor–like domain and S5–S6 form the pore domain, while TRP refers to the conserved C-terminal TRP domain located immediately downstream of S6, PH refers to the pore helix between S5 and S6, and LD refers to the ARD-S1 linker. The “Stereospecificity” column indicates whether a given binding site cluster shows preferential interaction with cholesterol over its stereoisomer epicholesterol. A value of “True” denotes clusters where interactions are stereospecific (i.e., cholesterol and epicholesterol exhibit distinguishable binding behavior at that site), whereas “False” indicates clusters where both sterols bind in a similar manner, suggesting a lack of stereospecific discrimination. This annotation reflects a qualitative comparison of binding patterns rather than an absolute exclusion of epicholesterol binding at those sites.

In the open-state channel, repeated cholesterol-binding clusters are predominantly located within helices S4–S6 (Figure 3). The most frequently observed cluster (Group 1; Table 2) involves residues from S5 and S6, identifying a lipid-accessible region near the central pore that may serve as a primary cholesterol interaction site. Additional clusters (Groups 2, 4, and 6) span the S1–S3 region, the S4–S5 linker, and adjacent hydrophobic grooves, consistent with cholesterol binding at intra- and interhelical crevices. A cytosolic/pre-TMD cluster (Group 5) incorporates N-terminal and TRP-helix residues, suggesting potential noncanonical cholesterol interactions that could stabilize the open state or modulate conformational transitions. Representative cholesterol poses from these clusters are illustrated in Figure 4, with van der Waals representations highlighting their locations relative to the protein surface and tetrameric symmetry.

**Figure 3:**
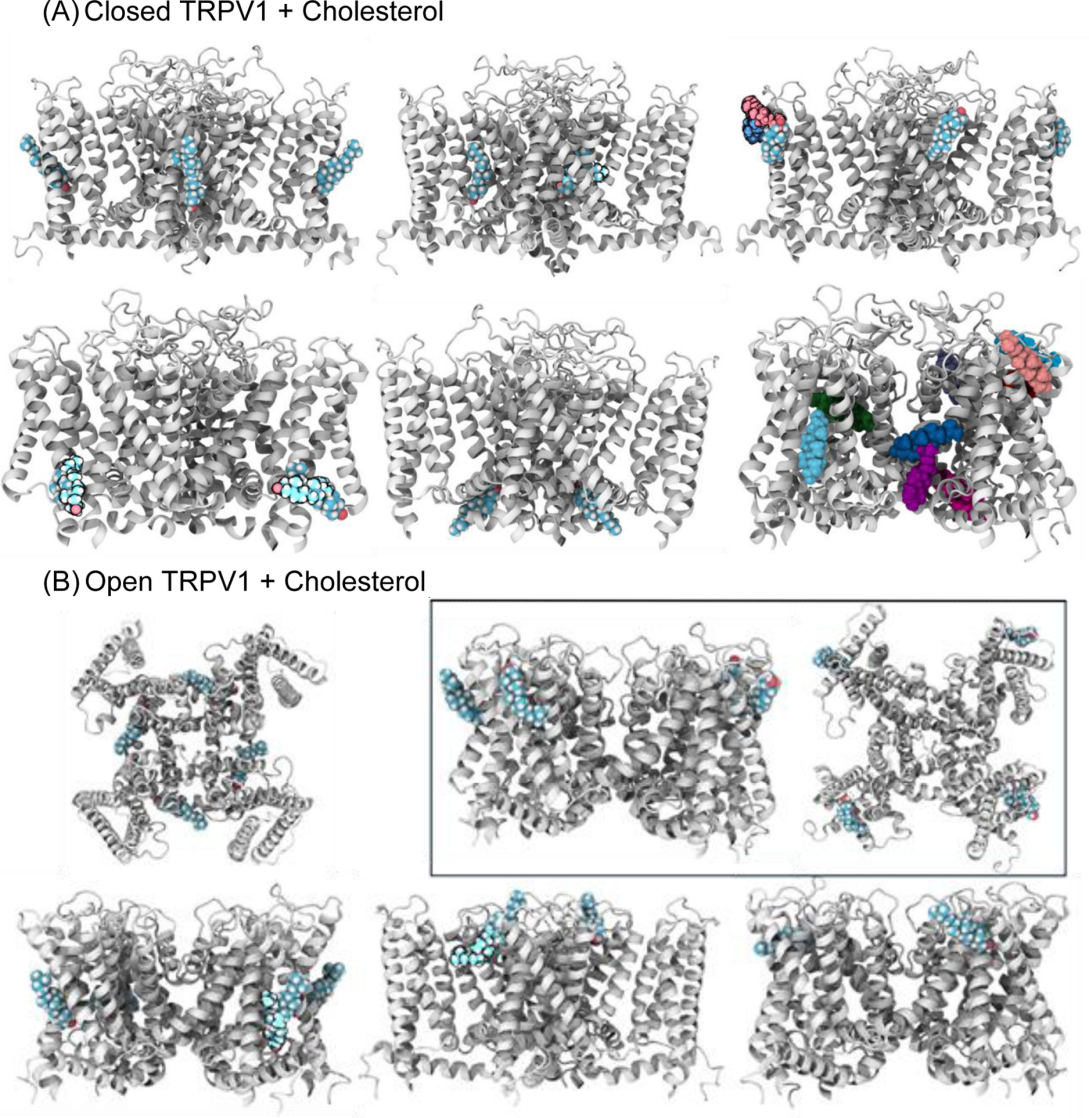
SILCS-identified cholesterol binding clusters in TRPV1. Cartoon representations of the closed (A) and open (B) TRPV1 structures are shown in white in new cartoon representation. Cholesterol molecules from representative SILCS-derived clusters are rendered in van der Waals representation, illustrating predicted interaction sites in both conformational states. Multiple equivalent binding solutions arise from the tetrameric architecture of TRPV1, with clusters recurring across subunits. When multiple conformations occurred at the same site, the van der Waals coloring was altered for visibility. The final structure of the closed conformation depicts all identified cholesterol-binding sites in this conformation, each shown in a different color.

**Figure 4:**
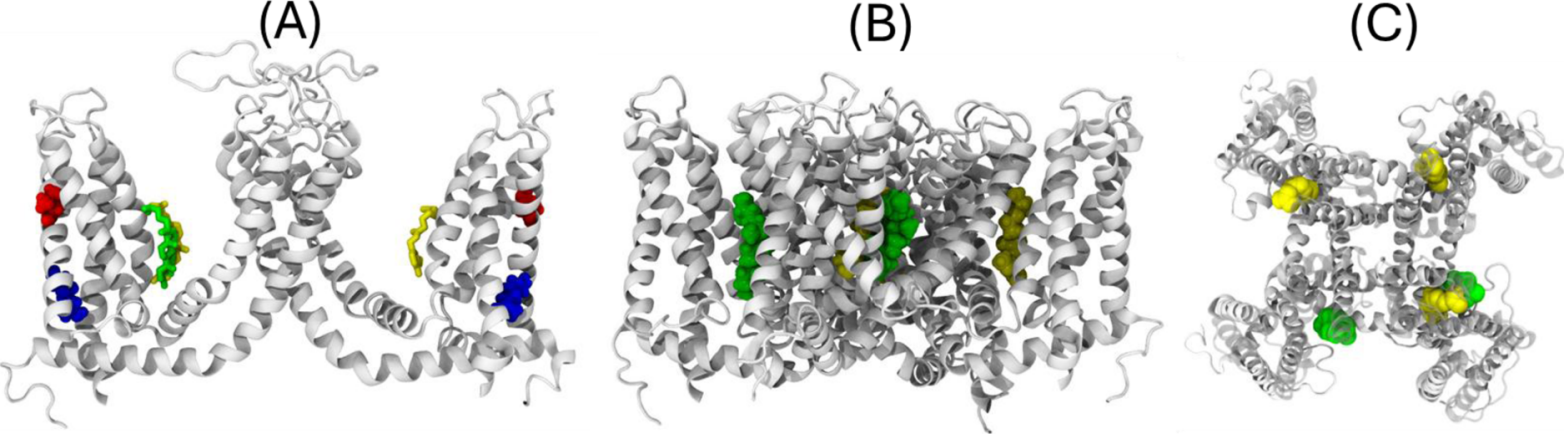
SILCS-predicted cholesterol pose at the capsaicin-binding site of open-state TRPV1. The TRPV1 ion channel in its open conformation is shown in white using a new cartoon representation. Capsaicin (yellow, licorice) is shown in its canonical binding pose, while cholesterol (green, licorice) is positioned according to the SILCS-derived pose identified in the closed-state TRPV1 structure that corresponds spatially to the capsaicin-binding site in the open conformation. (A) Side view showing two TRPV1 subunits. Residues V482 (red) and R491 (blue) are shown for reference. (B) Full tetrameric view with capsaicin (yellow) and cholesterol (green) shown in van der Waals representation. (C) Top-down view highlighting the relative positioning of cholesterol and capsaicin within the binding pocket. Together, these representations illustrate the SILCS-predicted overlap between ligand-binding regions across conformational states.

Analysis of the closed-state channel identifies both recurrent and conformation-specific cholesterol-binding sites. Several residues, including Val440, Tyr444, Ile573, Ser483, and Tyr487, are shared with the open-state clusters, suggesting core lipid-interaction regions accessible across gating states. Additional residues unique to the closed state, such as Tyr441, Thr476, Gly484, Phe488, Phe543, Ala546, and Thr550, form peripheral or transient pockets that may stabilize the closed conformation or reflect inhibitory roles for cholesterol (Table 2). Minor shifts in cluster composition, particularly around transmembrane and pre-TMD regions, highlight how lipid accessibility is dynamically coupled to protein conformational changes.

Interestingly, SILCS analysis predicts that cholesterol can occupy a binding pose overlapping the capsaicin site in the open-state TRPV1 (Figure 5). In this model, capsaicin is placed in its canonical binding orientation, while cholesterol is positioned according to the SILCS-derived pose originally identified in the closed-state structure. The vanilloid-binding pocket involves key residues critical for capsaicin binding, including Tyr511, Ser512, Met547, Asn551, and Lys571, which contribute hydrogen bonding and hydrophobic interactions. These results illustrate a structural overlap between cholesterol- and capsaicin-binding regions, suggesting that cholesterol may modulate TRPV1 activity by competing with vanilloid or stabilizing the vanilloid-binding pocket.

**Figure 5:**
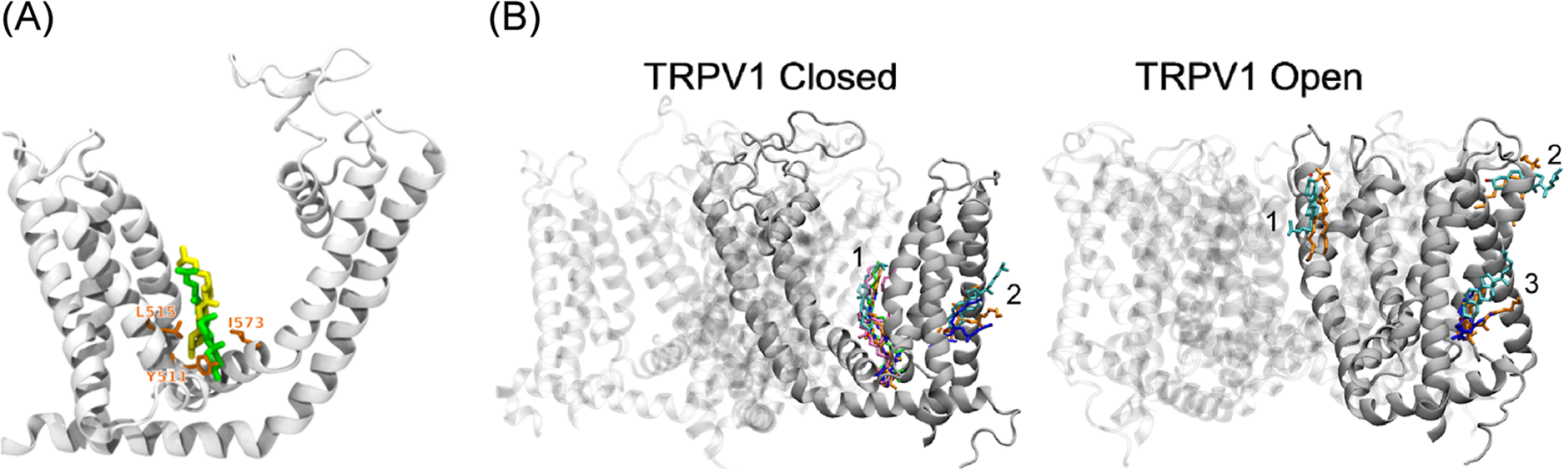
Comparison of SILCS predictions with experimentally resolved cholesterol-bound TRPV1 structures. (A) In PDB ID 8JQR (2024), crystallographic cholesterol (yellow) aligns with the SILCS hotspot (green), with interacting residues (orange) highlighted to confirm the binding site. (B) The closed and open states of TRPV1 showing SILCS hotspot locations for cholesterol aligned with lipids observed in crystallized structures. In the closed state, cluster 1 shows lipids LBN (orange) and 6O8 (blue) aligned with closed capsaicin hotspot 4 (cyan), while cluster 2 shows lipids XJ7 (orange), 6ES (blue), T7X (green), and 8IJ (pink) aligned with closed capsaicin hotspot 2 (cyan). In the open state, cluster 1 shows lipid 6OU (orange) aligned with open cholesterol hotspot 17 (cyan); cluster 2 shows lipid 6OE (orange) aligned with open cholesterol hotspot 42 (cyan); and cluster 3 shows lipids LBN (orange) and 6O8 (blue) aligned with open cholesterol hotspot 5 (cyan).

Across both open- and closed-state structures, the SILCS-derived clusters reveal a combination of recurrent and conformation-specific interaction sites. Tetrameric symmetry explains the repeated occurrence of equivalent binding clusters across subunits. The overlap with canonical CRAC/CARC motifs, together with alignment to functionally relevant regions such as the S4–S5-TRP domain and vanilloid pocket, underscores the biological significance of these interactions. Comparative analysis of open- and closed-state structures highlights how cholesterol accessibility and binding are dynamically influenced by conformational state, supporting a model in which TRPV1 regulation is mediated by both direct pore-proximal interactions and allosteric contacts at cytosolic and pre-TMD domains. The identified clusters (Table 2) define key cholesterol-interaction hotspots, providing a framework for understanding lipid modulation of TRPV1 function across gating states.

The SILCS approach was also applied to analyze epicholesterol interactions with TRPV1 in both open and closed conformations. Using epicholesterol as the ligand, SILCS-hotspots docking was performed on FragMaps generated for TRPV1, revealing clusters of residues mediating epicholesterol binding (Table S4, Figures S1 and S2). In the open-state channel, the most frequently observed clusters (Groups 1 and 4) involve residues from the S5–S6 helices and the S1–S3 region, highlighting lipid-accessible regions near the central pore as well as intra- and interhelical grooves. Additional clusters (Groups 2, 3, 5, and 6) span the S4–S5 linker, cytosolic/pre-TMD regions, and peripheral hydrophobic pockets, suggesting potential allosteric or stabilizing interactions. In the closed-state channel, epicholesterol-binding clusters include residues shared with the open state, such as Val440, Tyr444, Ser483, Tyr487, and Phe488 (Group 1), indicating core lipid-interaction hotspots accessible across gating states. Additional closed-state-specific clusters (Groups 3, 4, 5, and 6) involve residues in the S1–S2, S4–S5, and pore-forming domains, which may reflect conformation-dependent accessibility or regulatory roles for epicholesterol. The recurring clusters across subunits underscore the influence of TRPV1 tetrameric symmetry on ligand binding. Overall, these findings reveal both recurrent and conformation-specific epicholesterol interaction sites, emphasizing the dynamic nature of sterol modulation in TRPV1 and providing a framework for understanding stereochemistry-dependent lipid regulation.

Comparison of the SILCS-derived cholesterol and epicholesterol binding clusters identifies both shared (Figure S2) and distinct interaction sites, providing insight into the stereospecificity of sterol recognition by TRPV1. Several conserved residues, including Val440, Tyr444, Ser483, Tyr487, Ile573, Leu577, Cys578, Leu669, and Met581, are occupied by both cholesterol and epicholesterol across open- and closed-state structures, indicating core lipid-accessible hotspots within the S1–S3 and S5–S6 helices that likely accommodate sterols independent of their stereochemistry. Notably, epicholesterol also engages additional residues that are not prominent in cholesterol clusters, and vice versa, particularly in peripheral sites or cytosolic/pre-TMD regions, reflecting subtle stereochemical preferences and differences in binding pose orientations. These observations are consistent with the experimental use of epicholesterol as a tool to probe cholesterol specificity: while TRPV1 retains the capacity to interact with both sterols at key hotspots, the differential engagement of secondary sites highlights regions where stereochemistry may modulate binding affinity or functional effects. Collectively, this comparison supports a model in which TRPV1 contains a core set of sterol-recognition residues that accommodate cholesterol and sterol analogs, while secondary, conformation-dependent interactions contribute to the nuanced stereospecific regulation of channel activity.

Analysis of the epicholesterol cluster overlap between the open and closed states (Table S4) further highlights stereochemistry-dependent differences in sterol engagement. Among the open-state clusters, only one is shared with the closed-state, suggesting that epicholesterol explores additional binding sites preferentially accessible in the open conformation. Comparison with cholesterol clusters suggests that while several open-state clusters are common to both sterols (open epicholosterol Groups 1,2, 4 and 8), others are selectively occupied by either cholesterol or epicholesterol, reflecting differences in binding orientation or accessibility. In the closed state, cluster overlaps between epicholesterol and cholesterol are similarly partial, with three fully shared cluster and several sterol-specific clusters, implying that stereochemistry influences which residues are accessible or favored in a given conformation. Overall, this overlap analysis reinforces the concept of a core, conformation-independent set of sterol-interacting residues, supplemented by secondary, stereospecific sites that may modulate binding affinity or allosteric regulation depending on both sterol type and channel state.

To further support the findings from SILCS, TRPV1 crystallized structures containing lipids were compared to the SILCS findings. To our knowledge, two experimentally resolved cholesterol-bound structures are relevant for comparison. In the first structure (PDB ID: 8JQR), cholesterol was crystallized and aligns closely with the SILCS-predicted binding site. The correspondence between the SILCS hotspot and the crystallographic cholesterol is shown in Figure 5, and the interacting residues have been highlighted to show that this represents the same binding location discussed earlier.

The second structure (PDB ID: 8U2Z) captures TRPV1 in a dilated state that is distinct from both the open and closed conformations used in our simulations. No SILCS cholesterol pose directly overlaps with the crystallographic cholesterol in this case. Inspection of the exclusion maps indicates that this region was not sampled in the open conformation due to conformational constraints. However, the methanol oxygen FragMap aligns well with the cholesterol oxygen observed crystallographically, suggesting that this binding site may have been detected had the same protein conformation been used.

### Comparisons between the Detailed Classical Docking Studies and the SILCS-Derived Cholesterol Interaction Clusters

Cholesterol binding to TRPV1 in its open conformation identifies both structurally conserved and state-specific features, supported by convergent evidence from docking and SILCS analyses. Docking studies across PDBs 3J5Q, 3J5R, and 5IRX consistently identify the S4–S5 interface and intersubunit clefts involving helices S1, S4, and S5 as dominant cholesterol binding regions. Notably, residues such as Met552, Tyr555, Thr556 (S4) and Phe582, Val583, Leu585, Val586, Phe589 (S5) form recurring cholesterol interaction motifs (Sites 7a, 11a, 12a). These sites are topologically conserved and lie opposite the vanilloid pocket, highlighting their potential role in lipid-mediated gating regulation.

SILCS analysis of open-state TRPV1 identifies frequent cholesterol interaction clusters in overlapping regions, providing a more comprehensive and robust picture of sterol interactions than rigid-body docking. Group 1, comprising Ile573, Leu577, Met581, and Cys578 on or near S5 and the S4–S5 linker, corresponds closely to docking Sites 11*a*/12a, supporting the concept of a cholesterol-sensing zone at the S4–S5 *cis*-subunit interface. Additional docking-predicted intersubunit binding modes, including Phe438 and Phe439 (S1), Met552 and Thr556 (S4), and Val586 (S5), are partially mirrored in SILCS group 2, where residues such as Asn437, Tyr444 (S1), and Ile479 (S2) occur. Although SILCS does not highlight Phe438 or Met552 directly, the surrounding residues suggest a broadly permissive lipid-binding surface capable of accommodating sterol orientation variability, bilayer curvature, and dynamic protein motions that rigid docking cannot capture.

SILCS group 3 maps approximately to docking Sites 9*a*/10a. While the specific residues differ, both data sets implicate the intracellular face of the S2–S3 region as an auxiliary lipid interaction zone, reflecting conformational flexibility or differences in site accessibility.

SILCS group 4 in the open state aligns with sites 1a and 5a within normal docking, located between helices S1 and S2 at the extracellular membrane interface. The docking prediction shares the exact same residues as those shown in by SILCS. Interestingly, the docking predictions are within the closed state, whereas the SILCS solution is in the open state.

Importantly, SILCS further identifies additional cholesterol interactions not prominent in docking, such as groups 5 and 6, likely representing transient, low-occupancy, or allosteric sites that classical docking fails to detect.

Overall, these analyses highlight a core set of cholesterol interaction sites, particularly around S4–S5 and intersubunit clefts, that are robust across methods and conformations, while SILCS additionally captures secondary, conformation- and method-specific sites. Compared with classical docking, SILCS offers broader coverage of lipid-sensing surfaces, accounting for protein flexibility and solvent effects, making it a more reliable approach for mapping cholesterol interaction hotspots in TRPV1’s open state.

In closed-state TRPV1 (PDBs 3J5P, 5IRZ, and 5IS0), docking identified recurring sterol binding sites with distinct residue involvement. Dominant cholesterol sites include a primary site at the extracellular S1–S2 interface (Sites 1*a*/5a), the vanilloid binding pocket at the cytosolic boundary between subunits (Sites 1b, 3a, 5c), and a transmembrane pocket involving the S3 helix, S4–S5 linker, and TRP domain (Site 1c).

Comparison with SILCS clusters in open-state TRPV1 shows both convergence and divergence. SILCS group 1 aligns with docking Sites 3c (5IRZ) and 5d (5ISO) in the S4–S5-TRP domain, indicating a stable cholesterol hotspot across conformations. Group 2 overlaps with S1–S2 interface docking sites 5c and 11c highlighting this extracellular interface as a sterol binding region. Importantly, the other three sites in the closed SILCS docking did not strongly correlate with any of the classical docking sites. This suggests that they could be transient docking sites which are not detected by classical docking.

Together, these analyses indicate that the S1–S2 interface and the S4–S5-TRP domain region serve as primary cholesterol binding hotspots in TRPV1, consistently observed in both closed-state docking and open-state SILCS clustering. Variable occupancy of the vanilloid pocket underscores the dynamic nature of sterol modulation. By integrating SILCS FragMap analysis with classical docking and motif identification, we reveal multiple, symmetry-related cholesterol-binding sites in the TRPV1 tetramer, emphasizing their potential role in channel regulation and the functional impact of conformational context on sterol interactions. SILCS emerges as a superior method for robustly mapping lipid interaction landscapes, capturing both canonical and noncanonical binding regions with high fidelity.

### The Incidence of Cholesterol Binding to TRPV Channel: CRAC, CARC and CCM Motifs

Many studies have demonstrated that membrane cholesterol is a major regulator of ion channel function. Therefore, the identification of putative cholesterol binding sites is crucial for selective regulation. These sites include locations associated with the well-known cholesterol recognition amino acid consensus (CRAC) motif and its reversed form, CARC; he canonical CRAC motif is defined as -L/V-(*X*)^1–5^ -*Y*-(*X*)^1–5^ -R/K-, where (*X*) represents 1–5 residues of any amino acid. Due to the broad definition of CRAC motifs, they are ubiquitous in the TRPV family of ion channels. CARC motifs, which are inverted CRAC sequences with interchangeable tyrosine or phenylalanine residues, are even more frequent and have been shown to better predict cholesterol recognition in integral membrane proteins.

However, the ability of CRAC and CARC domains to predict actual cholesterol binding in large, multidomain proteins is limited. Only one of the seven CRAC motifs was identified to have all three constituents of its motif bound to by cholesterol, and only three of the seven CARC motifs were identified to have all three constituents of its motif bound to by cholesterol (Table 3). These observations likely reflect the distinct roles of motif residues: the leucine or valine contributes hydrophobic interactions, the tyrosine engages the sterol 3′–OH group, and the basic residue positions the motif at the membrane interface. Collectively, this suggests that interaction with all three residues is not required for sterol binding, and that lysine or arginine side chains primarily orient the motif via “snorkeling” into the aqueous environment.^40,44^

**Table 3:** Comparison of Cholesterol-Interaction Motif Detection in TRPV1^a^

Selection			SILCS		Classical Docking					
			3J5P	3J5Q	3J5P	5IRZ	5IS0	3J5Q	3J5R	5IRX
CRAC Motifs	V469–Y472–R474	469–474		4	1a		5a			
	V482–Y487–R491	482–491	1	2		3b	5d		9b	
	L524/V525/V527/V528–Y530–R534/K535	524–535	3	3						
	L553–Y555–R557	553–557			1b	3a	5c	7a	9b	11c
	L647–Y653–K656	647–656		6						
	L706–F712–K714/R717/K718	706–718								
CRAC-like Motifs	V482/V486–F488/F489–R491	482–491	1	2		3b	5d		9b	
	V527/V528/L529–F531–R534/K535	527–535	3	3						
	L706–F712–K714/R717/K718	706–718								
CARC Motifs	K431/R432–Y435–V440	431–440	4	2	1d	3b	5d	7a		
	R491–Y495–L497	491–497					5b		9a	
	R534/K535–Y537–V542	534–542								
	R579–Y584–V586/L588	579–588		1	1b		5a	7a		11b

a Summary of CRAC, CARC, and related motif detection across TRPV1 interaction sites in both open and closed conformations. Results from SILCS and classical docking approaches are presented, indicating the presence or absence of motifs at each site with the numbers indicating the corresponding cluster aligned with the motif.

To account for partial interactions, we evaluated clusters binding to the central aromatic residue and at least one of the other two residues. With this relaxed criterion, cholesterol clusters binding CARC motifs increased to ∼18%, and epicholesterol to ∼19%. CRAC motifs saw smaller increases (∼6% and ∼7%, respectively), reflecting their more restrictive definition.

The third established cholesterol-binding motif is the cholesterol consensus motif (CCM), which spans residues on adjacent helices: (W/*Y*)-(I/V/L)-(K/R) on one helix and (F/Y/R) on the second.

Conformation-dependent cholesterol interaction patterns.

To investigate the influence of protein conformation on cholesterol binding, we analyzed canonical and CRAC-like motifs in both open and closed TRPV1 states using SILCS FragMaps (Figure 6 and Table 3). Our results reveal a combination of recurrent, state-enhanced, and conformation-sensitive cholesterol interaction sites.

**Figure 6:**
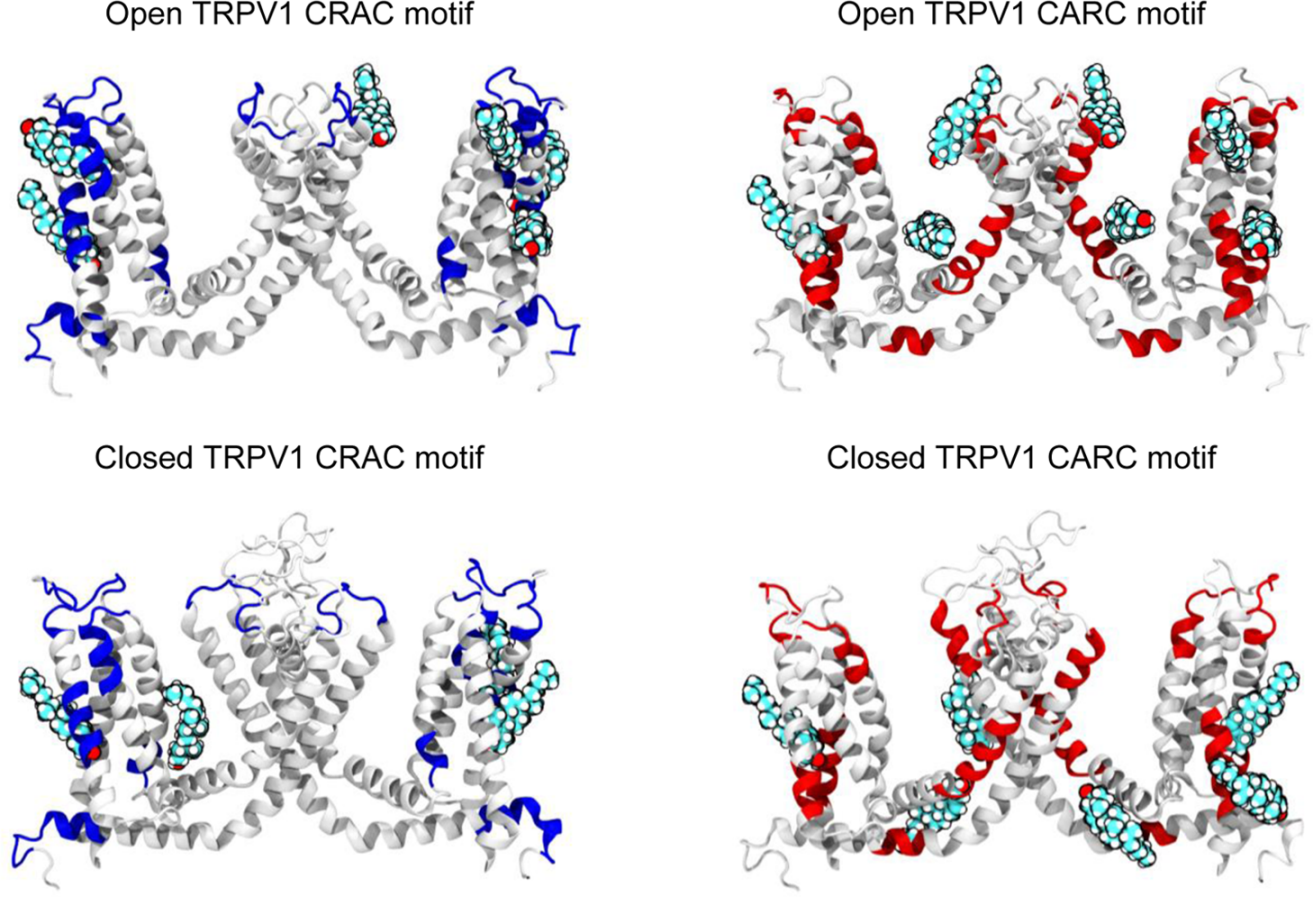
Cholesterol-binding motifs in the TRPV1 transmembrane domain. TRPV1 is shown in gray cartoon representation. The incidence of CRAC and CARC motifs is shown for cholesterol-binding clusters in the closed and open states. Cholesterol is displayed in van der Waals representation at predicted binding sites. The highlighted clusters map onto canonical cholesterol-recognition motifs with CRAC motifs in blue, and CARC motifs in red.

Conversely, specific motifs display pronounced conformation-dependent cholesterol interaction patterns. In the closed state, we observe enhanced cholesterol binding signals in regions such as Leu524 to Arg534, Leu553 to Arg557, and Leu725 to Lys735, indicating that protein closure exposes or structurally stabilizes these motifs, thereby facilitating cholesterol interaction. This behavior implies that cholesterol may play a role in stabilizing the closed conformation or that closure enhances cholesterol accessibility at these sites.

In contrast, motifs spanning Val482 to Arg491 show stronger cholesterol-interaction signals in the open state (see Figure 5 for the position of these residues), suggesting that this region becomes occluded or otherwise less favorable for cholesterol binding upon channel closure. This behavior may reflect dynamic cholesterol regulation during conformational transitions, with specific motifs being preferentially accessible in distinct structural states.

Several additional motifs, including Leu460 to Lys466, Val469 to Arg474, and Lys368 to Val377, displayed discontinuous or weak cholesterol interaction signals in both conformations, which may indicate low-affinity binding or transient, dynamic accessibility not strictly coupled to the open-closed transition Overall, our findings highlight the complex interplay between protein conformation and cholesterol accessibility. The data identifies both cholesterol interaction hotspots are likely essential for structural integrity, and state-specific motifs, which may contribute to cholesterol-dependent modulation of protein dynamics.

Overall, these findings highlight the complex interplay between protein conformation and cholesterol accessibility, identifying both structurally persistent hotspots and dynamic, state-specific motifs that may regulate channel function.

### LGFE Analysis of Cholesterol Binding

Comparing cholesterol binding in the open and closed conformations of TRPV1 is crucial to understand how conformational changes influence the accessibility and favorability of binding sites, as structural rearrangements may expose or occlude specific pockets.

We analyzed the predicted cholesterol binding landscape in TRPV1 using LGFE (Ligand Grid Free Energy) values computed from SILCS FragMaps for both open and closed TRPV1 conformations (Table 4). LGFE represents the sum of atomic grid free energy contributions for a ligand pose and serves as a relative indicator of predicted binding favorability, incorporating hydrophobic, polar, and hydrogen-bonding contributions. In the present work, LGFE values are used in their native, uncalibrated form as a comparative metric to rank ligand poses and binding sites based on predicted interaction quality. While LGFE scores can in principle be mapped to experimental binding free energies through offsets or Bayesian optimization approaches,^88^ such calibrations were outside the scope of this study.

**Table 4:** Predicted SILCs LGFEs of Cholesterol in TRPV1 Binding Sites^a^

conformation	cluster ID	# docking poses	LGFE (kcal/mol)
CLOSED	1	5	–11.32 ± 1.06
	2	3	–11.08 ± 0.92
	3	3	–9.54 ± 0.16
	4	4	–8.89 ± 0.86
	5	2	–5.95 ± 3.38
OPEN	1	4	–11.42 ± 0.71
	2	4	–10.11 ± 1.14
	3	4	–9.58 ± 0.49
	4	2	–7.38 ± 0.31
	5	2	–7.06 ± 0.14
	6	3	–4.97 ± 1.23

a Ligand grid free energy (LGFE, kcal/mol) values for cholesterol in closed and open TRPV1 conformations are shown. The data highlight site- and conformation-dependent differences. The number of SILCS docking poses should be four (one per monomer) if sampling is perfectly converged. At times, this number is lower than four because sampling was not perfect, and at other times, multiple similar poses occur in the same chain which can increase the number of poses past four.

Across closed-state clusters, LGFEs ranged from −12.9 to −3.6 kcal/mol, while open-state values ranged from −12.3 to −3.9 kcal/mol, with several clusters showing weaker (less negative) scores. Generally, more favorable (i.e., more negative) LGFE values in the closed state suggest that certain pockets may stabilize cholesterol binding, while the broad spread suggests that binding favorability varies across sites within each conformation. The SILCS-predicted LGFE values indicate that cholesterol binding is energetically favorable at identified sites in both open and closed TRPV1 conformations, with values ranging from approximately −10.0 to −11.5 kcal/mol. Analysis of spatial overlap between clusters identifies that one closed cluster (C1) coincides geometrically with one open cluster (O2), while one open cluster (O1) overlaps with one closed cluster (C2) (Figure 7). At these overlapping sites, the LGFE values indicate that binding is largely conserved: O1/C2 exhibit very similar energetics (−11.42 ± 0.71 vs −11.08 ± 0.92 kcal/mol), whereas C1/O2 shows a modestly less favorable binding in the open state (−11.32 ± 1.06 vs – 10.11 ± 1.14 kcal/mol), although the uncertainties partially overlap. These results suggest that cholesterol binding sites are mostly conserved in space between conformations, while small conformational changes associated with channel gating can subtly modulate their stabilization. Overall, the magnitude of the observed differences is comparable to the uncertainties, indicating that cholesterol binding is fine-tuned rather than gained or lost upon channel opening.

**Figure 7:**
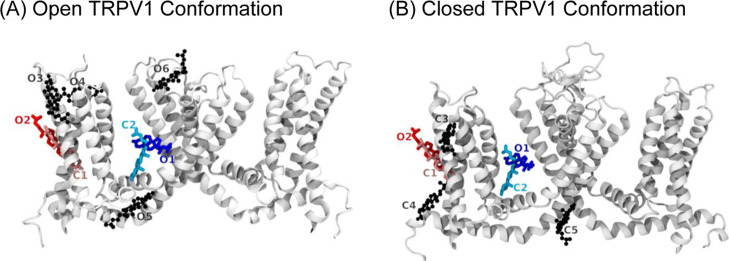
Comparison of SILCS-predicted cholesterol binding clusters in the (A) open and (B) closed conformations of the TRPV1 ion channel. SILCS dockings were analyzed to identify binding-site overlap between conformational states. Only two clusters in each state overlapped and are shown in red and blue. Clusters are labeled “O” (open) and “C” (closed). Open-state clusters without a corresponding closed-state cluster are shown in black (CPK representation). An analogous representation is shown for the closed conformation, illustrating that only a single overlapping binding location is present between states.

Interestingly, one of the cholesterol-binding sites also overlaps with a known capsaicin-binding pocket, suggesting that this location may serve as a hotspot for multiple ligands. The shared occupancy suggests that cholesterol could influence capsaicin access or binding stability, potentially modulating TRPV1 activation. This finding highlights the functional relevance of structurally conserved binding sites, where both endogenous (cholesterol) and exogenous (capsaicin) ligands may contribute to the fine-tuning of channel gating and overall TRPV1 function.

Together, these comparisons suggest that experimentally resolved cholesterol and lipid binding sites in TRPV1 are consistent with the SILCS-predicted hotspots, while also highlighting the importance of protein conformation in determining which sites are accessible.

## Conclusions

Cholesterol regulates TRPV1 channel activity both indirectly and directly. Indirectly, it upregulates TRPV1 expression by facilitating channel trafficking to the plasma membrane. Directly, cholesterol binds TRPV1 to inhibit capsaicin-induced activation, with stereospecificity demonstrated by the lack of similar inhibition by epicholesterol.

Capsaicin binds to TRPV1 at the well-characterized vanilloid binding site, a hydrophobic cavity formed at the interface of transmembrane helices. Docking studies show cholesterol frequently occupies this site in closed TRPV1 conformations, suggesting possible competitive inhibition. However, no significant difference in binding affinity between cholesterol and epicholesterol at this site was observed, limiting definitive conclusions. The vanilloid site is less accessible to cholesterol in open TRPV1 conformations, indicating conformation-dependent binding preferences.

Several potential allosteric cholesterol binding sites were identified, including extracellular ridges of S1/S2 helices, interfaces between pore-forming domains of adjacent subunits, and cytosolic termini of S1/S2. These sites, overlapping with previously reported annular lipid sites, exhibit subtle differences in cholesterol versus epicholesterol docking, suggesting a nuanced role in channel gating, although mechanistic details remain unclear.

Using SILCS fragment-based mapping, cholesterol binding clusters were mapped onto TRPV1’s open and closed states, revealing multiple cholesterol interaction motifs, including CRAC and CARC sequences. These clusters predominantly localize to transmembrane helices (S4–S6), consistent with cholesterol binding at intra- and interhelical grooves near the central pore. Additional clusters in cytosolic domains involve charged and polar residues, implying cholesterol may also modulate channel gating allosterically by interacting with intracellular regions.

Comparative analysis between open and closed states highlights both cholesterol-binding regions and state-specific sites. Core clusters are maintained across conformations, likely contributing to basal regulation, while unique sites in the closed state may stabilize the channel’s inactive form. This dynamic interplay suggests cholesterol binding modulates TRPV1 gating through a combination of direct pore-region and allosteric interactions. Overall, this comprehensive docking and motif analysis supports a model where cholesterol influences TRPV1 function through multiple, symmetry-related binding sites, involving both competitive and allosteric mechanisms modulated by channel conformation.

In the present study, cholesterol was identified in the hydrophobic cavity formed by S1–4 and the TRP domain in TRPV1. This cavity is in the membrane integral domain of all TRPVs, and thus, in contact with the plasma membrane, suggesting cholesterol and other lipids, would naturally fill the binding site. Indeed, lipid densities have been observed in this cavity for TRPVs.^16,17,19,20,89^ The identification of this site as a binding site for TRPV activating ligands, such as, 2-APB and 5′6′-EET, suggests a putative role of this binding site in the TRPV gating mechanism.^16,29,90^ Even if cholesterol is not found to activate TRPVs via this binding site, the near ubiquitous lipid occupation of this site suggests that cholesterol may stabilize TRPV channels by binding there. This is supported by the existence of a lipid density in this position in the TRPV1 structure bound to capsaicin^91^ and the existence of a cholesterol density within the same site from a more recent structure.^92^

While classical docking calculations are ideal for identifying multiple possible binding interactions between a protein and a ligand, the force fields and scoring algorithms employed suffer from well-known limitations.^29^ Besides, although ligand bonds are allowed to rotate around their torsional degrees of freedom, the rigid bonds and hard spheres used do not capture the true dynamic flexibility of the protein. Nevertheless, the high-throughput nature of docking simulations makes them an excellent starting point for determining potential binding sites and assessing the likely effect of a ligand on its target protein. The aim of the classical docking studies presented here was to quickly identify cholesterol and epicholesterol binding sites in the TRPV1 ion channel. The next step in characterizing these sites was to perform SILCS simulations of the channel embedded in a model membrane, allowing us to probe the energetic landscape of cholesterol interactions, account for the influence of the membrane environment on binding, and evaluate how the dynamic behavior of the protein may modulate cholesterol accessibility and affinity, features that could not be assessed using the classical docking approach.

Discovery of protein binding sites is an important milestone in the drug discovery timeline. With the binding site, a structure-based pharmacophore, that is, the steric and electronic features required to ensure the optimal supramolecular interactions with a specific biological target structure and to trigger or block a biological response, can be produced, to determine the spatial arrangement of specific functionalities required to elicit channel activation or inhibition.^93^ Due to the polymodality and wide expression in the body, either inhibition or activation of the TRPV family of ion channels could be desirable for the treatment of a specific condition in a specific cellular environment. TRPV channels have been implicated in several illnesses and thus, TRPV targeting pharmaceuticals are currently in the pipeline for the treatment of cancer, atherosclerosis and a plethora of nociceptive disorders.^63,94,95^ Our *in silico* analysis of multiple cryo-EM structures of the TRPV1 ion channel identifies dynamic and conformation-dependent cholesterol binding sites, predominantly localized at the vanilloid pocket and the S1–S2 interface. Across both closed and open states, cholesterol and its stereoisomer epicholesterol exhibit distinct but overlapping binding patterns influenced by subtle conformational variations and differences in hydroxyl group orientation. These findings highlight the vanilloid pocket as a conserved high-affinity sterol binding site and that cholesterol modulates TRPV1 function through multiple membrane-embedded and subunit-interface sites. This structural heterogeneity underscores the complexity of lipid–channel interactions and provides a molecular framework to better understand cholesterol’s regulatory role in TRPV1 gating and pharmacology.

Future work could employ advanced free energy methods, such as alchemical or potential-of-mean-force calculations, to obtain more accurate estimates of cholesterol binding affinities and to quantitatively rank the identified sites, building on the qualitative and energetic insights provided by SILCS. Complementary experimental validation could include site-directed mutagenesis of predicted interacting residues to assess functional effects on TRPV1 activity, or the use of photo switchable cholesterol analogs (*e.g.*, OptoChol1) to probe cholesterol occupancy and stereospecific effects in live-cell assays. These approaches would provide direct evidence linking the predicted binding sites to cholesterol-mediated modulation of TRPV1 function and could help refine pharmacological strategies targeting these channels.

## Supplementary Material



## Notes

Table S1. Overlap coefficients of selected SILCS FragMaps to assess convergence of simulations for the POPC/Chol system in closed and open TRPV1 conformations. Table S2. AutoDock-predicted cholesterol binding clusters, normalized cluster populations, mean binding energies, and interacting residues for closed and open TRPV1 structures. Table S3. AutoDock-predicted epicholesterol binding clusters, normalized cluster frequencies, mean binding energies, and interacting residues for closed and open TRPV1 structures. Figure S1. SILCS-identified epicholesterol binding clusters in closed and open TRPV1 conformations. Figure S2. Additional SILCS-identified epicholesterol binding clusters in closed and open TRPV1 conformations. Table S4. Conserved amino acid residue clusters mediating epicholesterol interactions in open and closed TRPV1 conformations identified from SILCS FragMaps and docking analyses.(PDF)
