# Supplementary material for: In Silico Identification of Cholesterol Binding Sites in the Transient Potential Receptor Vanilloid 1 (TRPV1) Ion Channel
Source: J Phys Chem B. 2026 Apr 13;130(16):4297–313. doi: 10.1021/acs.jpcb.5c08356 (PMC13112353; doi:10.1021/acs.jpcb.5c08356)
Supplement: Supplementary file 1 [file jp5c08356_si_001.pdf]

In-silico Identification Of Cholesterol Binding Sites In The Transient Potential Receptor Vanilloid 1 (TRPV1) Ion Channel

Jacob Morris[1*], Annabel SJ Eardley-Brunt[1*], Alexander J. Blayney[1], Carmen Domene[1**]

[1]Department of Chemistry, University of Bath, Claverdon Down, Bath BA2 7AY, United Kingdom

**Corresponding author: C.Domene@bath.ac.uk
*These authors contributed equally to this work

Table S1. Overlap coefficients of selected FragMaps indicating the extent of convergence of the SILCS simulations for the POPC/CHOL system. Overlap Coefficients (OC) were calculated as previously described in [1] between FragMaps calculated from simulation 1-5 and 6-10 from POPC systems. OC values >0.6 indicate satisfactory convergence. Fragmaps are listed for: methanol (MEOO), imidazole acceptor (IMIN), Imidazole nitrogen donor (protonated imidazole) (IMINH), formamide oxygen (FORO), methylannonium (MAMN), benzene (BENC), propane (PRPC), acetate carboxylate (ACEC), imidazole (GEHC), water (TIPO), formamide nitrogen (amide group) (FORN), acetamide oxygen (carbonyl of acetamide, used as an H-bond acceptor) (AALO), acetamide carbon (central carbon in acetamide) (AALC), and acetate carboxylate carbon, negatively charged carboxylate group (ACEC).

CLOSED TRPV1 conformation		OPEN TRPV1 conformation	
**FragMap**	**POPC**	**FragMap**	**POPC**
MEOO	0.76	MEOO	0.73
IMIN	0.74	IMIN	0.72
IMINH	0.74	IMINH	0.72
FORO	0.75	FORO	0.73
MAMN	0.75	MAMN	0.71
BENC	0.80	BENC	0.77
PRPC	0.78	PRPC	0.75
ACEC	0.79	ACEC	0.78
GEHC	0.83	GEHC	0.81
TIPO	0.96	TIPO	0.97
FORN	0.75	FORN	0.73
AALO	0.75	AALO	0.73
AALC	0.75	AALC	0.73
ACEC	0.73	ACEC	0.72

Table S2. AutoDock-predicted cholesterol binding clusters and binding affinities for TRPV1. Cholesterol-binding sites identified in both closed and open TRPV1 structures are listed along with their cluster percentages, mean binding energies (kcal/mol), and interacting residues. Cluster % indicates the fraction of docking poses within each cluster relative to all solutions. Since our analysis focused on the transmembrane domain (TMD), cluster percentages were normalized relative to the total number of docking poses in the TMD. Residue annotations in brackets denote the structural element containing each residue (*e.g.,* S1, S2–S3, S4–S5, S5, S6, TRP helix).

PDB ID	Site	Normalised Cluster %	Cluster %	Mean energy (kcal/mol)	Residues
3J5P	1c	5.21	3.33	-8,00	S505, F507 (S2-3). S510, Y511 (S3). R557 (S4-5). L574 (S4). I696, Q700 [H], I703, T704 (TRP).
	1d	3.64	2.33	-8.13	L506, F507 (S3). L574, R575, C578, R579, F582 (S5). F559, Q561, M562 (S4-5).
	1a	3.64	2.33	-8.22	L443, Y444, I447, A450, A451, Y454 (S1). F473, R474, G477, L480 (S2).
	1a	28.14	18.00	-8.35	I447, A450, A451, Y454 (S1). G470, F473, R474, T476, G477 (S2).
	1d	4.69	3.00	-8.39	F438 (S1). T556 (S4). F559, Q561, M562 (S4-5). L506, F507 (S2-3A). L574, R575, C578, F582 (S5A).
	1a	6.25	4.00	-8.54	I447, A450, A451, Y454 (S1). G470, F473, R474, T476, G477 (S2).
	1b	5.21	3.33	-8.73	L515 (S3). M547, T550, N551, L553, Y554 (S4). E570, I573 (S4-5). F587 (S5A). L669 (S6A).
	1b	3.64	2.33	-8.54	L515 (S3). A546, M547, T550, L553 (S4). A566 [H], I569, E570, I573 (S4-5). F587, F591 (S5A). L669 (S6A).
	1b	5.21	3.33	-8.79	L515 (S3). T550, N551, L553, Y554 (S4). A566, E570, I573 (S4-5). L669 (S6A).
	1c	13.54	8.66	-9.31	S510, S512 (S3). Y554, R557 (S4). V567, E570, K571 (S4-5). I696, Q700 (TRP).
	1c	3.13	2.00	-9.67	L506, F507, D509, Y511 (S3). A566, V567, E570, K571, L574 (S4-5). I696, Q700 (TRP).
	1c	4.69	3.00	-9.86	Y487, F488, R491 (S2). S512, E513, F516 (S3). Y555, R557 (S4). I703 (TRP).
	1b	6.77	4.33	-9.99	L515 (S3). M547, T550, L553, Y554, R557 [H] (S4). A566, E570, I573 (S4-5). F587, F591 (S5A). L669 (S6A). Q700 (TRP).
	1b	6.25	4.00	-9.93	L515 (S3). M547, T550, L553, Y554, R557 (S4). A566, E570, I573 (S4-5). F587 (S5A). L669 (S6A). Q700 (TRP).
5IRZ	3b	2.77	2.33	-7.36	N437 [H], V440, Y444 (S1). L480, G484, Y487, F488 (S2).
	3b	2.38	2	-7.44	Y453, Y454, R455 [H] (S1). R474 (S2). V596 [H], T597 (S5A). I599, E600, N628 (PA).
	3b	4.36	3.66	-7.42	N437 [H], V440, Y444, I447 (S1). L480, G484, Y487 (S2).
	3c	2.38	2	-7.5	N437, V440, Y441, L443, Y444, I447 (S1). L480, G484, Y487, F488, R491 (S2). Y555 (S4).
	3a	3.57	3	-7.88	S512 (S3). T550, N551, Y554, R557 (S4). F587 (S5A). L669 (S6A).

	3a	16.27	13.66	-8.00	L515 (S3). M547, T550, N551, L553, R557 (S4). A566, I569, E570 (S4-5). Q700 (TRP). F587 (S5A). L669 (S6A).
	3a	5.55	4.66	-8.09	Y511, L515 (S3). L553, Y554, R557 (S4). A566, I573 (S4-5). Q700 [H] (TRP). L669 (S6A).
	3a	44.85	37.66	-8.88	L515 (S3). A546, M547, T550, L553, Y554, R557 [H] (S4). A556, I569, I573 (S4-5). Q700 (TRP). F591 (S5A). L669 (S6A).
	3a	17.86	15.00	-8.79	L515 (S3). M547, T550, L553, Y554, R557 [H] (S4). A566 (S4-5). F591 (S5A). A665, L669 (S6A).
5ISO	5e	3.11	2.33	-6.37	C442 (S1). W549, M552 (S4). L577 [H], C578, M581, F582, L585, V586, F589 (S5A).
	5d	2.67	2.00	-7.14	N437, V440, Y441, L443, Y444, I447 (S1). L480, Y487, F488 (S2). Y555 (S4-5).
	5b	5.78	4.33	-7.27	Y489, G492, I493, F496 (S2). L506, S510 (S2-3). I514, F517, L521 (S3).
	5b	11.11	8.33	-7.35	G492, Y495, F496, R500 (S2). S502, L503 (S2-3). E513 [H], I514, F517 (S3).
	5d	5.78	4.33	-7.5	N437, V440, L443, Y444 (S1). L480, S483, G484, Y487, F488 (S2).
	5d	3.55	2.66	-7.55	N437, V440, L443, Y444 (S1). L480, S483, G484, Y487, F488 (S2).
	5a	13.34	10.00	-7.97	I447, A450, A451, Y454 (S1). G470, F473, R474, T476, G477, L480 (S2).
	5a	42.24	31.66	-8.05	I447, A450, A451, Y454 (S1). G470, F473, R474, T476, G477, L480 (S2).
	5c	4.88	3.66	-8.64	Y511, S512, L515 (S3). M547, T550, L553 [H], Y554 (S4). E570, I573 (S4-5). F591 (S5A). A665, L669 (S6A).
	5c	7.55	5.66	-9.2	Y511, L515 (S3). M547, T550, L553, T556, R557 (S4). A566, V567, E570, I573 (S4-5). F587 (S5A). L669 (S6A).
3J5Q	7b	2.34	2.00	-7.88	K603, S632, L635, K639 (P). D646, L647, E648, F649 (PA). K656 (S6A).
	7a	5.86	5.00	-7.77	I446 (S1). W549, M552,T556 [H] (S4). F582, V583, V586, F589 (S5A).
	7a	32.42	27.66	-8.09	F439, C442 (S1). W549, M552, L553 (S4). F582, V583, L585, V586 (S5A).
	7a	9.38	8.00	-8.05	F438, F439, C442, I446 (S1). W549, M552, L553 (S4). L585, V586, (S5A).
	7a	6.63	5.66	-8.1	F438, I446 (S1). W549, M552, L553, T556 (S4). F582, V583, V586, F589 (S5A).
	7a	2.73	2.33	-8.36	F438 (S1). W549, M552, T556, F559 (S4). F582, L585, V586, F589 (S5A).
	7a	4.69	4.00	-8.33	Y435, F438 (S1). M552, Y555, T556, F559 (S4). F582, L585, V586, F589 (S5A).
	7a	35.94	30.66	-8.26	F438 (S1). W549, M552, L553, T556 (S4). M562, Y565 (S4-5). F582, V583, L585, V586, F589 (S5A).
3J5R	9c	3.24	2.33	-7.60	M568, K571, M572, R575 (S4-5). L681, E684 [H], T685 (S6).
	9a	19.91	14.33	-8.12	Y495, F496, R499, R500 (S2). S502, L503, L506, F507, V508, D509, S510 (S2-3). I514 (S3).
	9a	33.80	24.33	-8.13	Y495, F496, R499, R500 (S2). S502, L503, L506, F507, V508, D509, S510 (S2-3).
	9a	21.30	15.33	-8.29	Y495, F496, R499, R500 (S2). S502, L503, L506, F507, V508, D509, S510 [H] (S2-3).
	9a	6.95	5.00	-8.15	Y495, F496, R499 (S2). S502, L503, L506, F507, V508, D509 (S2-3). I514 (S3).
	9a	4.17	3.00	-8.55	Y495, F496, R499, R500 (S2). S502, L503, L506, F507, V508, D509 (S2-3).
	9b	2.78	2.00	-9.51	R491, G492, Y495 (S2). D509, S510 (S2-3). S512, E513, F516 (S3). Y554 (S4). Q700, I703 (TRP).

	9b	7.86	5.66	-9.75	R491, G492, Y495 (S2). D509 [H], S510 (S2-3). S512, E513, F516, F517 (S3). Y554 (S4). Q700, I703 (TRP).
5IRX	11c	2.72	2.33	-7.31	F507, Y511, L515 (S3). F543, A546, M547, T550 (S4). L574 (S5). F587, F591 (S5A). L669 (S6A).
	11c	3.11	2.66	-8.13	Y511, S512, L515 (S3). M547, T550, N551, L553, Y554 (S4). I569, I573 (S5). A665, L669 (S6A).
	11a	21.02	18.00	-7.94	M552, Y555, T556, F559 (S4). F582, L585, V586, F589 (S5A).
	11a	47.87	41.00	-8.04	M552, Y555, T556 (S4). F582, L585, V586, F589 (S5A).
	11b	2.34	2.00	-7.95	M581, Y584, L585, L588 (S5). Y631, C634, L635, F638, Y641, I642 (P). L664, I668 (S6A).
	11a	5.44	4.66	-8.40	M552, Y555, T556 (S4). F582, L585, V586, F589 (S5A).
	11b	17.51	15.00	-8.79	L577, M581, Y584 (S5). L635, F638, T641, I642 (P). L675, L678 (S6). L664, I668 (S6A).

Table S3 AutoDock-predicted epicholesterol binding clusters and binding affinities for TRPV1. Epicholesterol-binding sites identified in both closed and open TRPV1 structures are listed along with their cluster frequencies, mean binding energies (kcal/mol), and interacting residues. Cluster frequency indicates the fraction of docking poses within each cluster relative to all solutions. Since our analysis focused on the transmembrane domain (TMD), cluster frequencies were normalized relative to the total number of docking poses in the TMD. Residue annotations in brackets denote the structural element containing each residue (*e.g.*, S1, S2–S3, S4–S5, S5, S6, TRP helix).

PDB ID	Site	Normalised Cluster %	Cluster %	Mean energy (kcal/mol)	Residues
3J5P	2b	3.59	2.33	-7.67	F543, A546, M547, T550 (S4). I573, L577 (S4-5). F591 (S5A). L662, A665, I668, L669 (S6A).
	2c	5.64	3.66	-7.95	L506, F507 (S2-3). Y511 (S3). L574, R575, C578, R579, F582 (S5). M562 (S4-5A).
	2d	4.10	2.66	-7.90	G558, F559, Q560, Q561, M562 (S4-5). R575, C578, R579 (S5A).
	2a	13.33	8.66	-8.10	A450, A451, Y454 (S1). G470, F473, R474, T476, G477, L480 (S2).
	2a	4.62	3.00	-8.14	I447, A450, A451, Y454 (S1). G470, F473, R474, T476, G477 (S2).
	2a	7.70	5.00	-8.12	I447, A450, A451, Y454 (S1). G470, D471, F473, R474 (S2).
	2c	11.79	7.66	-8.34	L506, F507, D509, S510 (S2-3). Y511, S512 [H] (S3). R557 (S4). E570, L574 (S4-5).
	2a	3.59	2.33	-8.40	L443, I447, A450, Y454 (S1). F473, R474, G477, L480 (S2).
	2a	4.62	3.00	-8.31	A450, Y454 (S1). P456, V463, K466 (S1-2). G470, D471 [H], F473, R474 (S2).
	2c	3.59	2.33	-8.14	L506, F507 (S2-3). R575 [H], C578, R579 (S5). F559, Q560, Q561 (S4-5A). R701 (TRP).
	2a	7.17	4.66	-8.38	I447, Y454 (S1). G470, F473, R474, G477, L480 (S2).
	2b	10.78	7.00	-8.33	S512, L515 (S3). A546, M547, T550, N551, Y554 (S4). I573 (S4-5). F587, F591 (S5A). A665, L669 (S6A).
	2c	3.08	2.00	-9.45	F488, R491, Y495 (S2). S510 (S2-3). S512, E513, F516 (S3). R557 (S4). Q700, I703 (TRP).

	2b	9.75	6.33	-10.58	L515 (S3). M547, T550, L553, Y554 (S4). A566, E570, I573 (S4-5). F587 (S5A). L669 (S6A).
	2b	6.67	4.33	-10.56	L515 (S3). T550, L553, Y554, R557 (S4). A566, E570, I573 (S4-5). F587 (S5A). L669 (S6A).
5IRZ	4b	2.61	2.00	-7.10	V440, Y441, L443, Y444, I447 (S1). L480, G484, Y487, F488 (S2).
	4a	3.91	3.00	-7.40	Y511, L515 (S3). M547, T550, L553 (S4). A566, I569, I573, L574 (S4-5). F587 (S5A). L669 (S6A).
	4a	2.61	2.00	-7.83	L515 (S3). T550, L553, R557 (S4). A566, I573 (S4-5). L669 (S6A).
	4a	3.47	2.66	-7.95	S512, L515 (S3). T550, L553, Y554, R557 (S4). A566, I569 (S4-5). F587 (S5A). L669 (S6A).
	4a	7.38	5.66	-8.49	S512, L515 (S3). M547, T550 [H], L553, R557 (S4). A566, I569 (S4-5). L669 (S6A).
	4a	19.57	15.00	-8.80	L515 (S3). A546, M547, T550, N551, L553, R557 (S4). A566, E570 [H] (S4-5). Q700 (TRP). F591 (S5A). A665 (S6A).
	4a	41.75	32.00	-8.86	L515 (S3). A546, M547, T550, N551, L553, R557 (S4). A566, E570 (S4-5). Q700 (TRP). F591 (S5A). A665, L669 (S6A).
	4a	18.70	14.33	-8.40	L515 (S3). M547, T550 [H], L553, Y554, T556, R557 (S4). G563, A566, V567 (S4-5). Q700 (TRP). F587 (S5A). L669 (S6A).
5ISO	6c	4.69	3.33	-6.65	C442 (S1). W549, M552 (S4). L577 [H], C578, M581, F582, L585, V586, F589 (S5A).
	6c	3.75	2.66	-6.70	C442 (S1). W549, M552 (S4). L577 [H], C578, M581, F582, L585, V586, F589 (S5A).
	6e	3.28	2.33	-6.72	Y489, G492, I493, Y495, F496 (S2). L506, S510 (S2-3). I514, F517, L521 (S3).
	6d	2.82	2.00	-6.96	M547, T550, L553 (S3). I661, A665, L669 (S6A).
	6e	4.23	3.00	-7.23	N437, V440, Y441, L443, Y444, I447 (S1). L480, G484, Y487, F488 (S2). Y555 (S3).
	6a	5.64	4.00	-7.46	A450, Y454 (S1). G470, F473 [H], R474 (S2).
	6b	3.28	2.33	-7.72	V440, Y441, L443, Y444, I447 (S1). L480, S483, G484, Y487, F488 (S2).
	6b	6.10	4.33	-7.61	N437 [H]x2, V440, L443, Y444, I447 (S1). L480, Y487, F488, R491 (S2).
	6b	2.82	2.00	-7.39	N437 [H], V440, L443, Y444, I447 (S1). G484, Y487, F488, R491 (S2).
	6a	42.27	30.00	-8.09	I447, A450, A451, Y454 (S1). G470, F473, R474, T476, G477 (S2).
	6a	17.84	12.66	-8.06	I447, A450, A451, Y454 (S1). G470, F473, R474, T476, G477, L480 (S2).
	6d	3.28	2.33	-8.10	Y511, L515 (S3). M547, T550, L553, T556, R557 (S4). A566, V567, E570, I573 (S4-5). L669 (S6A).
3J5Q	8	10.69	8.66	-8.26	F439 [H], C442, I446 (S1). W549, L553 (S4). V583, L585, V586 (S5A).
	8	45.69	37.00	-8.35	F438, F439 [H], C442, L443, I446 (S1). W549, M552, L553, T556 (S4). V586 (S5A).
	8	5.75	4.66	-8.35	F438 (S1). W549, M552, L553, T556 (S4). M562 (S4-5). F582, V583, L585, V586, (S5A).
	8	14.82	12.00	-8.45	F438, I446 (S1). W549, M552, L553, T556 (S4). F582, V583, L585, V586, F589 (S5A).
	8	16.05	13.00	-8.15	F438 (S1). W549, M552, T556 (S4). F559, M562 (S4-5). F582, V583, L585, V586 (S5A).
	8	6.99	5.66	-8.58	F438, M442, I446 (S1). W549, M552, L553 [H], T556 (S4). F582, V583, L585, V586, F589 (S5A).
3J5R	10b	6.44	5.00	-7.63	Q561, I564, M568 (S4-5). M568, K571, M572, R579 (S4-5A). L681, M682, T685, I689 (S6A).
	10a	5.58	4.33	-8.19	Y495, F496, R499 (S2). S502, L503, L506, F507, V508, D509, S510 (S2-3).
	10a	11.59	9.00	-8.19	Y495, F496, R500 (S2). S502, L503, L506, F507, V508, D509, S510 (S2-3). I514 (S3).

	10a	4.29	3.33	-8.34	Y495, F496, R499, R500 (S2). S502, L503, L506, F507, V508, D509, S510 (S2-3). I514 (S3).
	10a	15.88	12.33	-8.35	Y495, F496, R499, R500 (S2). S502, L503, L506 [H], V508, S510 (S2-3). I514 (S3).
	10c	3.86	3.00	-8.49	L577, F580, M581, Y584, L585, L588 (S5). C634, F638 (P). I660, L664, I668, I672, L673 (S6A).
	10a	6.86	5.33	-8.69	Y495, F496, R499, R500 (S2). P501, S502, L503, V508, D509 [H], S510 (S2-3). I514 (S3).
	10a	45.50	35.33	-8.89	Y493, Y495, F496, L497, R500 (S2). S502, L503, F507 [H], V508, D509 (S2-3).
5IRX	12a	2.71	2.33	-7.68	F438 (S1). W549, M552 [H], L553, Y555, T556 (S4). F582, L585, V586, F589 (S5A).
	12a	3.09	2.66	-7.85	W549, M552, Y555, T556 (S4). F559, M562 (S4-5). F582, L585, V586, F589 (S5A).
	12a	14.35	12.33	-7.83	M552, L553, T556 (S4). M562 (S4-5). F582, V583, L585, V586, F589 (S5A).
	12a	3.09	2.66	-8.17	Y511, L515 (S3). F543, M547, T550, N551, L553 (S4). I569, I573 (S4-5). F587 (S5A). L669 (S6A).
	12a	7.75	6.66	-8.22	M552, T556 (S4). F582, L585, V586, F589 (S5A).
	12a	44.98	38.66	-8.08	F438 (S1). M552, T556 (S4). F582, L585, V586, F589 (S5A).
	12a	3.09	2.66	-8.19	Y435 [H], F438 (S3). M552, L553, Y555, T556 (S4). F559, M562 (S4-5). F582, L585, V586 (S5A).
	12a	3.09	2.66	-8.28	Y511, S512, L515 (S3). F543, M547, T550, N551, L553, Y554, R557 (S4). I569, I573 (S4-5). F587, F589 (S5A). A665, L669 (S6A).
	12b	12.80	11.00	-8.63	L577, F580, M581, Y584, L585, L588 (S5). Y631, L635, F638 (P). L664 (S6A).
	12b	5.04	4.33	-8.83	L577, M581, Y584 (S5). F638, T641 (P). L675 (S6). I661 [H], L664, I668 (S6A).

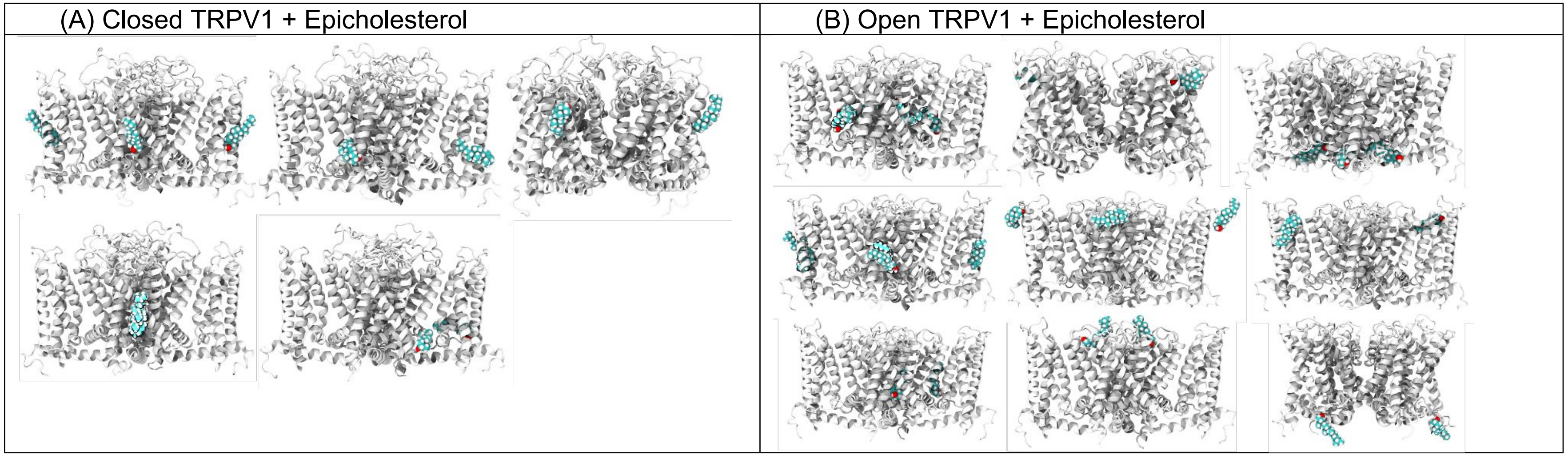

Figure S1. *SILCS-identified epicholesterol binding clusters in TRPV1.* Cartoon representations of the closed (A) and open (B) TRPV1 structures are shown in white in new cartoon representation. Epicholesterol molecules from representative SILCS-derived clusters are rendered in van der Waals representation, illustrating predicted interaction sites in both conformational states. Multiple equivalent binding solutions arise from the tetrameric architecture of TRPV1, with clusters recurring across subunits.

(A) Closed TRPV1 + Epicholesterol

(B) Open TRPV1 + Epicholesterol

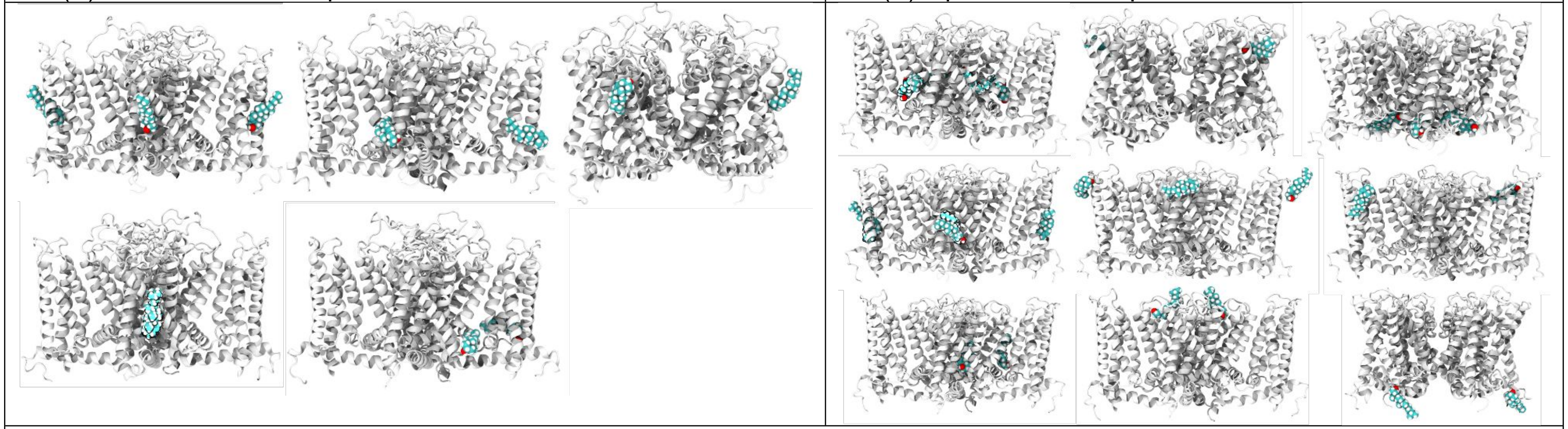

Figure S1. *SILCS-identified epicholesterol binding clusters in TRPV1.* Cartoon representations of the closed (A) and open (B) TRPV1 structures are shown in white in new cartoon representation. Epicholesterol molecules from representative SILCS-derived clusters are rendered in van der Waals representation, illustrating predicted interaction sites in both conformational states. Multiple equivalent binding solutions arise from the tetrameric architecture of TRPV1, with clusters recurring across subunits.

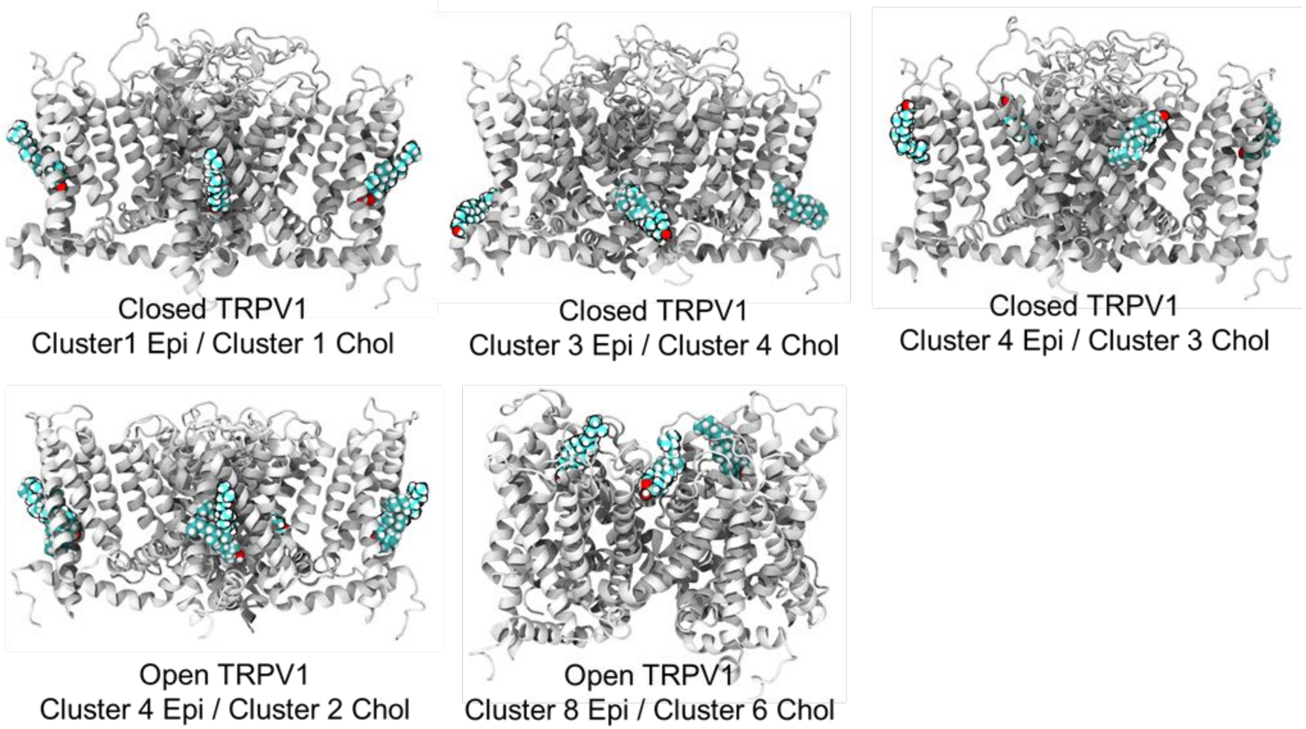

Figure S2. SILCS-identified epicholesterol binding clusters in TRPV1. Cartoon representations of the closed (A) and open (B) TRPV1 structures are shown in white in new cartoon representation. Epicholesterol molecules from representative SILCS-derived clusters are rendered in van der Waals representation, illustrating predicted interaction sites in both conformational states. Multiple equivalent binding solutions arise from the tetrameric architecture of TRPV1, with clusters recurring across subunits.

Table S4. Conserved clusters of amino acid residues mediating epicholesterol interactions in the open and closed conformation of TRPV1 ion channel. Clusters of overlapping residues were identified by analyzing 58 and 66 epicholesterol-protein clusters of interactions respectively derived from SILCS FragMaps and epicholesterol docking. Each cluster corresponds to a group of residues of the protein where one or more atoms of an amino acid is at 3 Å of an epicholesterol molecule. Only residues shared across all entries within each cluster are reported, representing conserved regions potentially mediating cholesterol binding. The occurrence column indicates the number of independent cholesterol-binding entries contributing to each cluster. The "Stereospecificity" column indicates whether a given binding site cluster shows preferential interaction with cholesterol over its stereoisomer epicholesterol. A value of "True" denotes clusters where interactions are stereospecific (*i.e.*, cholesterol and epicholesterol exhibit distinguishable binding behavior at that site), whereas "False" indicates clusters where both sterols bind in a similar manner, suggesting a lack of stereospecific discrimination. This annotation reflects a qualitative comparison of binding patterns rather than an absolute exclusion of epicholesterol binding at those sites.

Group	# Protein Subunits Occupied	Residues	Stereospecificity
		**OPEN TRPV1**	
1	4	Ala665 Cys578 Ile573 Ile668 Leu577 Leu669 Met581 Thr550	False
2	2	Arg474 Glu478 Ile479 Met523 Met541 Phe448 Ser481 Tyr530 Tyr537 Val482 Val527	False
3	3	Ala690 Gln560 Glu684 Ile564 Leu681 Lys694 Met568 Ser693 Val686	True
4	3	Asn437 Leu443 Leu480 Phe488 Tyr444 Tyr487 Val440	False
5	3	Ile479 Leu465 Tyr472 Val475	True
6	2	Ala539 Arg534 Glu536 Leu529 Phe543 Ser540 Val525 Val658	True
7	2	Ala546 Ala566 Ala665 Arg557 Cys578 Glu570 Ile569 Ile573 Ile661 Ile668 Leu662 Leu669 Leu673 Met547 Met581 Phe543 Phe587 Phe591 Ser512 Thr550 Tyr511	True
8	2	Ala657 Ile660 Leu635 Lys656 Phe649 Thr650 Tyr631	False
9	2	Asn393 Glu397 Leu334 Lys238 Lys332 Ser342 Tyr401 Val398	True
		**CLOSED TRPV1**	
1	3	Val440 Tyr441 Tyr444 Thr476 Leu480 Ser483 Gly484 Tyr487 Phe488	False
3	2	Arg432 Ile433 Phe436 Phe438 Phe439 Tyr435	True
4	2	Leu524 Met523 Ser532 Val482 Val486 Val527 Val528	False
5	1	Phe490 Ser483 Tyr487 Val486	False
6	2	Leu503 Phe496 Phe507	True
